# Sulfur‐Stabilized High Entropy Oxysulfides Enable Efficient C—C Bond Cleavage in Ethylene Glycol Electrooxidation for Sustainable Plastic Upcycling to Formate

**DOI:** 10.1002/cssc.202502529

**Published:** 2026-03-03

**Authors:** Saikat Bolar, Akitaka Ito, Chunyu Yuan, Meiyi Wang, Akira Yamaguchi, Masahiro Miyauchi, Takeshi Fujita

**Affiliations:** ^1^ School of Engineering Science Kochi University of Technology Kami City Kochi Japan; ^2^ Department of Materials Science and Engineering School of Materials and Chemical Technology Institute of Science Tokyo Meguro Tokyo Japan

**Keywords:** ethylene glycol electrooxidation, high‐entropy oxysulfides, high‐entropy oxides, plastic waste upcycling

## Abstract

The conversion of plastic waste into value‐added chemicals coupled with energy‐efficient H_2_ production is a sustainable strategy for addressing environmental and energy challenges, for example, the selective electrooxidation of polyethylene terephthalate (PET)‐derived ethylene glycol (EG) to C1 products via C—C bond cleavage is important for advancing PET electro‐reforming. Herein, a high‐entropy transition metal oxysulfide is synthesized through the one‐step room‐temperature incorporation of S into a high‐entropy transition metal oxide and evaluated as a catalyst for EG electrooxidation under alkaline conditions. Covalently bound S stabilizes O vacancies and high‐valence transition metal states through defect‐induced charge redistribution, enhancing lattice stability and promoting the establishment of an advantageous electronic structure. The developed catalyst is highly active, selective, and durable, achieving efficient C—C bond cleavage and formic acid production with a faradaic efficiency of 84.6%. S incorporation enhances both lattice‐oxygen and adsorbed‐oxygen mechanism pathways for C—C bond cleavage and accelerates hydrogen atom transfer, thereby enabling concerted formate formation. Replacing the anodic O_2_ evolution reaction with EG electrooxidation markedly reduces the required cell voltage, highlighting the high–entropy oxysulfide's promise as an electrocatalyst for plastic upcycling and energy–efficient green H_2_ generation.

## Graphical abstract

Room‐temperature synthesis method of high‐entropy oxy‐sulfide (HEOS) is introduced. Compared to high‐entropy oxides (HEO), sulfur incorporation promotes oxygen vacancy stabilization and facilitates C—C bond cleavage, improving efficiency of ethylene glycol electrooxidation to formic acid. Lower cell overall voltage enables sustainable plastic upcycling and green hydrogen generation, showcasing catalytic performance through tailored defect engineering and electronic modulation over HEO.

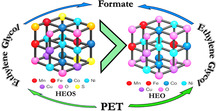

## 1 Introduction

H_2_ is a promising carbon‐free energy carrier and fundamental feedstock for the chemical industry. Given that the major H_2_ production methods, such as the steam reforming and gasification of fossil fuels, result in substantial CO_2_ emissions, sustainable alternatives are urgently required [1, 2, 3]. One of such alternatives is water electrolysis, which, however, has a moderate energy efficiency, relies on expensive noble‐metal catalysts, and therefore features a limited economic feasibility. Consequently, cost‐effective strategies for renewable energy‐powered H_2_ production from net‐zero carbon sources are highly sought after.

The overall overpotential of electrochemical water splitting can be reduced by replacing its primary energy‐intensive step the sluggish four‐electron anodic O_2_ evolution reaction (OER) with the oxidation of small organic molecules having oxidation potentials lower than that of water [4, 5, 6]. This strategy not only increases the energy efficiency of water splitting but also enables the use of cheap catalysts comprising earth‐abundant elements. Biomass‐derived products or organic waste, such as ethanol, glycerol, ethylene glycol (EG), and sugars, present an opportunity for the production of H_2_ with a net‐zero carbon footprint. The electrochemical reforming of these organics can generate valuable chemicals, which makes the overall process more profitable and reduces the dependency on fossil resources [4, 5]. EG holds promise as a liquid fuel because of its low toxicity and high power density (112 mW cm^−2^), theoretical energy density (7.56 kWhL^−1^), and boiling point (198°C) [7, 8, 9]. Compared with its methanol‐based counterpart, the direct EG fuel cell exhibits a higher theoretical capacity and operational flexibility over a broader temperature range because of the high boiling point of EG [10, 11]. As the smallest polyol, EG readily accessible via the chemical industry or degradation of polyethylene terephthalate (PET) can be considered a model reactant for investigating the selective electrooxidation of alcohols. The electrooxidation of EG (≈0.6 US$ kg^−1^) is a promising route to value‐added C1/C2 products (≈3.1 US$ kg^−1^) [12, 13, 14]. Formic acid (formate in alkaline medium), a key feedstock in chemical and pharmaceutical industries, can be produced via the electrocatalytic oxidation of methanol in alkaline media [15, 16]. This method is more economically advantageous than traditional high‐temperature and high‐pressure processes because of its sustainability, efficiency, and environmental benefits.

Owing to the slow natural degradation of plastics (e.g., PET), their excessive use and inadequate recycling pose a substantial environmental threat [17]. Therefore, systematic plastic recycling is essential from societal, economic, and environmental perspectives. PET is widely used in packaging and textile production, with its global consumption in 2020 estimated as 7297.7 kilotons of virgin PET resin and 1189.4 kilotons of recycled PET resin across 41 countries [18, 19, 20]. Currently, PET packaging waste is largely disposed of through landfill incineration, although a part of this waste can be more reasonably utilized (in) directly, and a part is scattered on beaches. Thus, excessive PET consumption can cause serious environmental pollution if not handled properly.

Fortunately, PET is readily hydrolyzed into EG and terephthalic acid (PTA), and the thus produced EG can be electrochemically converted into useful products to make the reuse of PET economically viable. Therefore, PET‐derived EG is a valuable feedstock for the sustainable production of H_2_ and generation of valuable chemicals, which can facilitate compliance with the solar energy requirements for overall water splitting.

EG oxidation (EGOX) holds promise for value‐added chemical production, direct fuel cells, and PET recycling [18, 19]; however, the feasibility of its realization depends on the efficiency and product selectivity of the EG oxidation reaction (EGOR). The EGOR involves complex multibond cleavage and multielectron transfer processes and therefore has to be promoted by efficient, stable, and selective electrocatalysts. Current research on EGOR catalysts primarily focuses on (i) the design and synthesis of advanced catalysts with high activity, stability, and selectivity, achieved by favoring the C1 pathway or optimizing C2 product yields while minimizing undesirable C2 byproduct formation and (ii) the investigation of EGOR mechanisms to deepen our mechanistic understanding of this reaction and enable rational catalyst design [20, 21].

Electrochemical EGOX plays a pivotal role in the conversion of waste‐derived or industrially available EG into valuable chemicals, such as formate, glycolic acid, oxalic acid and so on. Traditional EGOR catalysts contain noble metals (e.g., Pt and Pd) and have been optimized through alloy formation, structural modification, and substrate integration [21]. However, the high cost and limited supply of noble metals hinder the commercialization of these catalysts. Consequently, nonprecious‐metal catalysts, particularly transition metal oxides, phosphides, and sulfides, have emerged as promising alternatives because of their high electrocatalytic activity, selectivity, and stability [21].

Considerable attention has been drawn to the electrocatalytic oxidation of alcohols, particularly that promoted by noble metal–based catalysts [22]. Jang et al. realized efficient electrochemical EGOX using Pt nanoparticles supported on Se‐doped porous carbon, achieving a maximum EG conversion of 94.6% and glycolic acid (GCA) selectivity of 84.4% [23]. Electrochemical EGOX at Co and Ni electrodes in alkaline media was reported to yield value‐added chemicals, with the C1 pathway favoring formate (≈60% selectivity on Ni) and C2 pathway favoring glycolate (≈43% selectivity on Co at 1.60 V vs. the reversible hydrogen electrode (RHE)) [24]. A single‐Pt‐atom catalyst supported on TiO_2_ selectively produced glycolate and formate with a combined faradaic efficiency of 99.7% in an alkaline medium, which was ascribed to strong metal–support interactions [25]. Electrochemical EGOX on Pt and Pd electrodes in alkaline media yielded glycolic acid (selectivity > 85%), whereas Rh and Ir electrodes showed minimal activities, primarily producing glycolate and oxalate [26]. Similarly, Pt nanoparticles supported on Se‐doped porous carbon demonstrated an outstanding catalytic performance, achieving an EG conversion of 94.6% and a GCA selectivity of 84.4%, which underscores the efficacy of heteroatom‐doped carbon supports in enhancing activity and product selectivity [27]. NiSe was reported to efficiently catalyze EGOX (faradaic efficiency > 80%), favoring the formation of oxalate via glycolate [28].

High‐entropy alloys (HEAs) have emerged as promising next‐generation catalysts because of their tunable elemental compositions and adjustable stoichiometries [29]. These alloys form solid solutions with a random distribution of constituent elements because of entropic interactions, with each element functioning as an independent catalytic center [29, 30]. The atomic arrangement in HEAs markedly influences their selectivity for multi proton/‐electron transfer reactions. Despite these advantages, the vast number of possible elemental combinations and inherent complexity of HEAs hinder rational catalyst design, optimization, and modeling [29].

Herein, we present a room‐temperature high‐entropy oxysulfide (HEOS) that enables the electrocatalytic recycling of waste PET by facilitating the selective oxidation of EG to formate in an alkaline medium (KOH) while minimizing the overpotential of water electrolysis. The conversion of PET‐derived EG into formate offers substantial environmental and economic advantages, given that formic acid is widely utilized in pharmaceutical, textile, and leather industries, as well as an antibacterial agent in livestock feed. Additionally, formic acid holds promise as a high‐energy‐density fuel for direct formate fuel cells and an efficient liquid carrier of H_2_ enabling safe and scalable H_2_ storage and transportation [31].

Electrochemical EGOX plays a crucial role in sustainable H_2_ production, notably reducing the energy input required for overall water splitting. Unlike conventional thermochemical processes, which operate under high‐temperature and high‐pressure conditions, EGOX‐coupled water splitting occurs at ambient temperature, offering the benefits of an enhanced cost efficiency and environmental sustainability. Furthermore, this catalytic approach facilitates plastic waste upcycling, supporting the transition toward a circular carbon economy while reducing dependence on fossil‐derived feedstocks.

By leveraging the catalytic properties of HEOS and its precursor (high‐entropy oxide, HEO), we optimize the anodic oxidation pathway, improving both efficiency and stability with clear mechanistic insight. As a catalyst for the EG‐to‐formate conversion, the defect‐rich undercoordinated HEOS outperforms HEO in terms of reaction kinetics and selectivity. This precise and controlled conversion of EG to formate opens new avenues for EGOX chemistry, expanding its applicability across industrial electrochemical processes.

## 2 Results and Discussion

### 2.1 Synthesis and Physicochemical Properties

**FIGURE 1:**
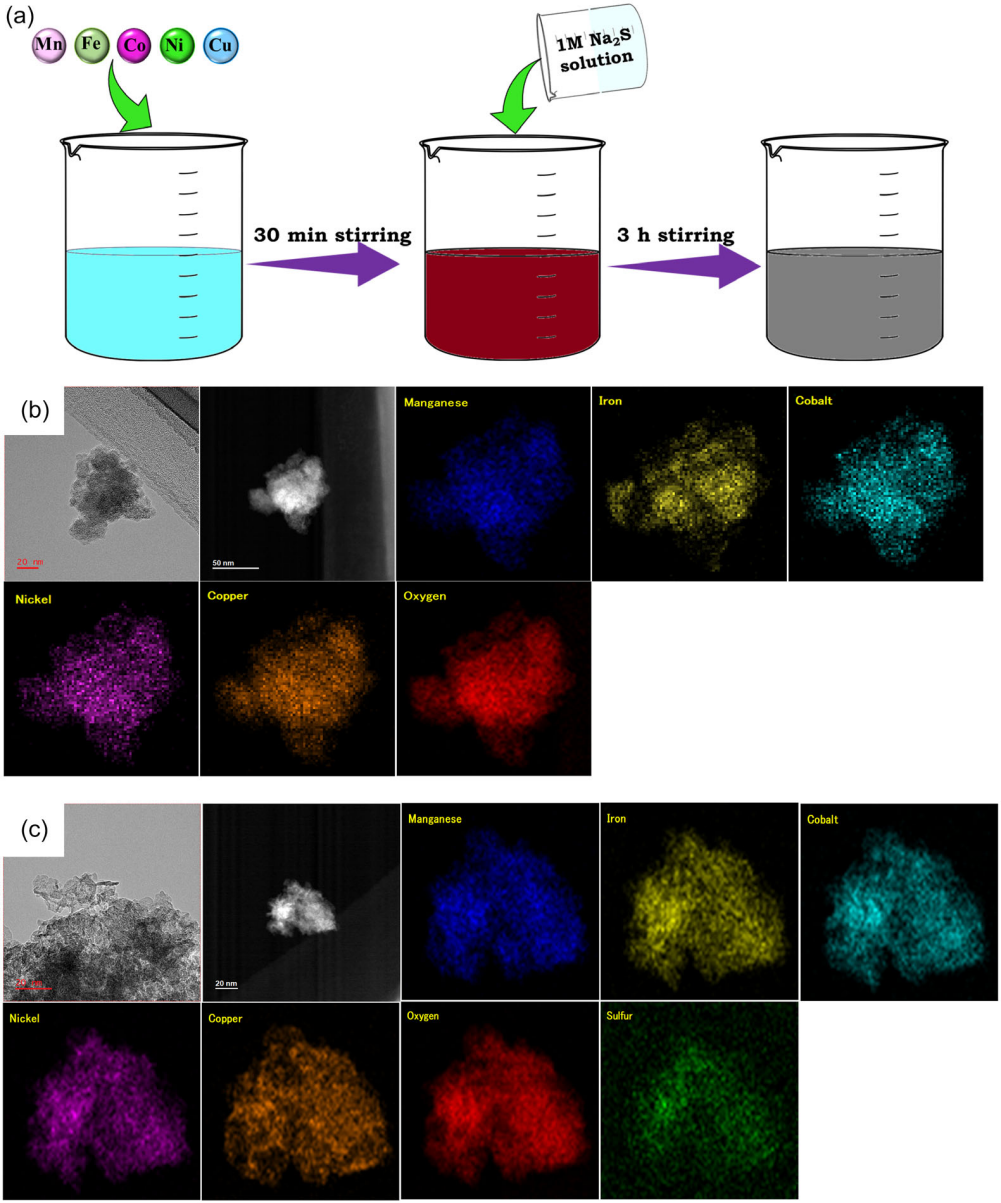
(a) Schematic room‐temperature synthesis of the high‐entropy oxysulfide (HEOS). High‐resolution transmission electron microscopy and STEM‐EDX images of the (b) high‐entropy oxide (HEO) and (c) HEOS.

HEOS was synthesized by treating an aqueous solution containing Mn, Fe, Co, Ni, and Cu chlorides with Na_2_S upon stirring under ambient conditions (Figure 1a). This approach represents an advancement in room‐temperature HEO synthesis, incorporating modifications that enhance phase stability and compositional uniformity [30, 32]. The selection of Mn, Fe, Co, Ni, and Cu was inspired by the composition of the Cantor alloy, in which case Cr was replaced with Cu to preserve the equi‐atomic distribution while leveraging the chemical versatility of Cu (Figure 1b,c) [33]. High‐resolution transmission electron microscopy and scanning transmission electron microscopy–energy‐dispersive X‐ray spectroscopy (STEM‐EDX) analyses confirmed the formation HEO and HEOS and demonstrated the uniform distribution of constituent elements therein. The corresponding selected area electron diffraction patterns and high‐resolution transmission electron microscopy images revealed the amorphous structure of these phases (Figures S1 and S2).

The constituent elements were selected because their closely spaced electronegativities (Mn: 1.55, Fe: 1.83, Co: 1.88, Ni: 1.91, Cu: 1.90; Pauling scale) suppressed strong preferential bonding and facilitated homogenous atomic distribution [34], thus mitigating phase segregation and ensuring homogeneous microstructures (Tables S1 and S2). The high oxidation potential of Cu substantially contributed to the electrochemical stability of HEOS. The interplay between the most noble (Cu) and most electrooxidation‐active (Mn) incorporated metals enhanced corrosion resistance, while Mn and Fe contributed to the formation of stable oxide layers. Co and Ni played a critical role in the stabilization of the face‐centered cubic (FCC) phase, suppressing intermetallic segregation and enhancing mechanical robustness. Ni also improved oxidation resistance, minimizing the susceptibility of HEO and HEOS to environmental degradation (Figure S3, Tables S1 and S4).

The elemental distribution in HEOS was more uniform than that in HEO, which was ascribed to S forming weaker bonds with transition metals than O and thus facilitating dynamic atomic rearrangement during synthesis [35]. Furthermore, S‐rich environments promoted the uniform diffusion of metal cations, whereas in HEO, the stronger bonding with O could lead to the localized clustering of certain elements (Tables S1 and S2) [36]. Within the employed reaction environment, sulfide anions exhibit relatively positive electrochemical potentials (*E* ^o^(S/S^2−^) = −0.447 V versus the normal hydrogen electrode), that is, are readily oxidized in aqueous media [37].

As a result, S‐centered redox processes can occur at potentials similar to those of metal‐centered redox processes. S is larger and softer than O, and M—S bonds display higher degrees of covalency than M‐O ones [38]. Therefore, from a structural viewpoint, the large and diffuse 3p orbitals of S engage in weaker *π*‐interactions with transition metal centers, which leads to bridging being preferred to terminal interactions. This bridging behavior arises because the delocalized electron density of S facilitates the distribution of its bonding interactions across multiple metal atoms (as opposed to forming a single strong bond with one metal center). This property is particularly relevant for HEOSs, in which case S enhances atomic mixing and element dispersion uniformity by engaging in multimetal coordination (Figures S4 and S5). Additionally, S‐rich environments promote the uniform diffusion of metal cations, further stabilizing the bridging interactions. In contrast, O, with its smaller and more localized 2*p* orbitals, forms stronger terminal bonds, which leads to the localized clustering of elements in HEOs. The collective electrocatalytic activity of individual elements, along with their structural and bonding characteristics, results in a cocktail effect, with synergistic interactions among the transition metals enhancing catalytic efficiency and thereby enabling selective and energy‐efficient EGOX.

X‐ray diffraction (XRD) analysis (Figure S3) revealed a mixed‐phase structure comprising FCC configurations, attributed to the minimal lattice strain resulting from the similar atomic radii of Mn (127 pm), Fe (126 pm), Co (125 pm), Ni (124 pm), and Cu (128 pm) [39]. The HEO pattern exhibited broad peaks (2*θ* = 37°, 44°, and 63°) characteristic of an FCC lattice, suggesting the formation of a single‐phase solid solution comprising transition metal (Mn, Fe, Co, Ni, and Cu) oxides. These broad peaks indicated a semi crystalline oxide phase that probably formed because of entropy‐driven stabilization [40]. The HEOS pattern displayed markedly broadened and weakened peaks indicative of amorphous structure. Upon sulfur incorporation, HEOS exhibits a markedly different diffraction envelope, including pronounced broad features around ≈20° and ≈35° (2*θ*), indicating substantial modification of short‐range ordering and lattice coherence. These changes are attributed to partial anion substitution (O^2−^ → S^2−^), which introduces local distortion and alters metal‐anion coordination environments, thereby reshaping the XRD intensity distribution and reflecting the formation of a structurally more disordered oxysulfide lattice The introduction of S into the oxide matrix induced substantial lattice distortion because of the radius of S^2−^ (184 pm) exceeding that of O^2−^ (140 pm), disrupting the long‐range ordering of the crystal lattice and facilitating the formation of a disordered metal oxysulfide solid solution stabilized by a high configurational entropy [41]. The transition from the semi crystalline oxide to the predominately amorphous oxysulfide (Figure S6) highlights the considerable impact of anion substitution on the crystallinity, lattice dynamics, and phase stability of high‐entropy materials. Raman spectroscopy further corroborates the anionic structure evolution revealed by XRD. Compared to HEO, HEOS exhibits an additional low‐frequency band at ≈300 cm^−1^ attributed to M–S vibrations, along with a pronounced downshift of the oxygen‐related M–O mode from ≈610 to ≈445–450 cm^−1^ [42, 43]. This shift arises from sulfur incorporation into the metal–anion framework, where substitution of O^2−^ by the larger and more polarizable S^2−^ induces local lattice distortion, weakens neighboring M—O bonds, and leads to phonon softening (Figure S7). The mixed‐anion (O/S) coordination combined with high‐entropy disorder results in asymmetric features of transition‐metal oxysulfides, confirming the formation of a genuine oxysulfide lattice rather than a physical mixture of oxide and sulfide phases.

**FIGURE 2:**
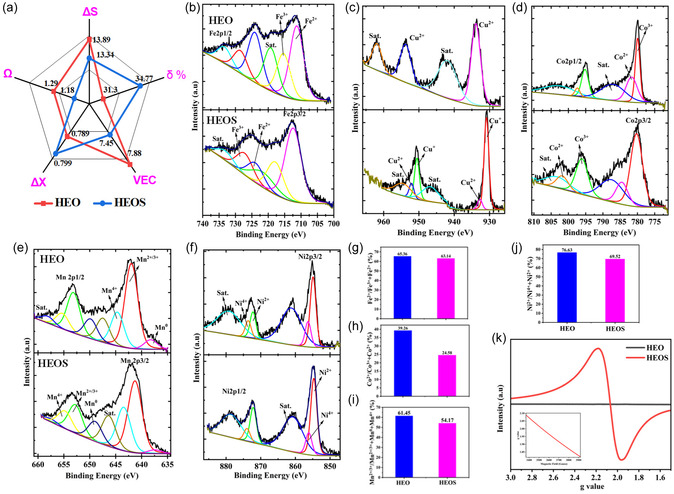
(a) Thermodynamic parameters of HEOS and HEO indicating the enhanced tendency for vacancy formation in the former material. Deconvoluted (b) Fe 2*p*, (c) Cu 2*p*, (d) Co 2*p*, (e) Mn 2*p*, and (f) Ni 2*p* X‐ray photoelectron spectra of HEOS and HEO. (g–j) Fractions of selected Mn, Fe, Co, and Ni oxidation states in HEO and HEOS prior to electrochemical oxidation. (k) Electron paramagnetic resonance spectra of HEO and HEOS.

The thermodynamic and structural characteristics of HEO and HEOS were analyzed to elucidate the influence of S incorporation on phase stability and structural complexity. The calculated atomic size mismatch (*δ*) of HEO (31.3%) and HEOS (34.8%) notably exceeded the typical threshold for single‐phase solid solution formation in HEAs, indicating that both systems were unlikely to form simple FCC or BCC structures (Figure 2, Tables S3–S5). Instead, the large atomic size disparities, primarily due to the high O and minimal S contents, were expected to drive the formation of complex multiphase microstructures. In HEO, the substantial O content (36.9 at%) probably stabilized metal oxide phases (e.g., MnO, NiO, CuO) or spinel‐type structures (e.g., (Mn, Fe, Co)_3_O_4_) [44, 45, 46, 47]. HEOS, featuring a lower O content and higher S content than HEO, was anticipated to exhibit a more diverse phase assemblage comprising oxides, minor sulfide phases (e.g., MnS and CuS), and potentially residual metallic solid solutions formed by Mn, Fe, Co, Ni, and Cu [48].

Despite the high Ω values (1.29 for HEO and 1.18 for HEOS), which exceeded the critical value of 1.1 and suggested the thermodynamic feasibility of high‐entropy configurations, the combined effects of the large δ and electronegativity differences (Δ*χ* = 0.789 for HEO and 0.799 for HEOS) disfavored the stabilization of simple solid solutions (Figure 2a, Tables S3–S5). The valence electron concentrations (VEC) of HEO (7.88) and HEOS (7.45) fell within the intermediate range, suggesting a potential tendency for the formation of FCC‐like metallic phases [30]. However, owing to the dominance of O and S, more complex ionic structures (e.g., rock‐salt, spinel, or perovskite‐type ones) reflecting the governing role of O coordination and ionic bonding were expected. The VEC of HEOS was minimally lower than that of HEO, indicating a reduced likelihood of FCC‐like metallic phases and favoring the formation of diverse oxide and sulfide structures. The configurational entropies of HEO (1.67 R (R = universal gas constant)) and HEOS (1.60 R) confirmed the high‐entropy nature of both materials, reinforcing their phase stability and suitability for functional applications [2, 35]. Compared with HEO, HEOS featured a lower mixing entropy despite containing more elements. This counterintuitive result was ascribed to the uneven elemental distribution of HEOS, where S was present at a markedly lower content (≈4.08 at%) than the other elements. The incorporation of this minimal amount of S disrupted the compositional uniformity of HEOS, thereby diminishing its configurational entropy. This behavior highlights the fact that the magnitude of the entropy gain in high‐entropy materials is governed not only by the number of elements but also by their atomic contents [2, 35, 46, 47]. Overall, the interplay between the configurational entropy, atomic size mismatch, VEC, and electronegativity differences endowed HEO and HEOS with electronic and structural characteristics that were expected to enhance electrochemical performance by enabling a heterogeneous active surface and tailored electronic states.

The results of X‐ray photoelectron spectroscopy (XPS) analysis indicated that anion engineering via sulfuration markedly altered the electronic structure of the Mn–Fe–Co–Ni–Cu high‐entropy lattice in HEOS. Survey spectra (Figure S8) revealed that HEO and HEOS featured uniform elemental distributions. Upon the partial replacement of lattice O with S, the Fe 2p, Co 2p, and Cu 2*p* peaks (Figure 2b–d) shifted to higher binding energies, indicating enhanced charge‐transfer polarization in HEOS. As S is less electronegative than O, M—S bonds display greater covalency than M—O ones, withdrawing electron density from metal centers and stabilizing higher oxidation states [49, 50].

In contrast, the Mn 2*p* peaks of HEOS (Figure 2e) were located at lower binding energies than those of HEO, suggesting partial reduction (Mn^3+^/Mn^4+^ → Mn^2+^) and an overall electron density gain due to the strong affinity of Mn for the softer S atoms [51]. The Cu^+^ state is rarely stabilized in oxides but was favored in HEOS because of its high‐entropy lattice and soft Cu–S coordination environment [52]. The mixed Ni^2+^/Ni^0^ states, which are highly covalent, showed negligible binding‐energy shifts and, hence, minimal electronic‐structure changes (Figure 2f). These element‐specific trends collectively demonstrate that sulfuration enhanced the covalency of metal–ligand bonds, redistributed charge across the constituent metals, and selectively stabilized reduced states (Mn^2+^, Cu^+^), increasing the diversity of catalytically active sites [52, 53].

The fractions of selected oxidation states derived from XPS data (Figure 2g–j) indicated that S incorporation favored the formation of high‐valent surface cations (Co^3+^, Fe^3+^, Cu^2+^, Mn^3+^/Mn^4+^), which was attributed to the synergistic effects of enhanced covalency and configurational entropy within the high‐entropy lattice [54, 55]. The electron paramagnetic resonance (EPR) spectrum of HEOS (but not HEO) featured a pronounced signal (*g* = 2.05) characteristic of electron‐trapped O vacancies, confirming that sulfuration increased the density of lattice defects (Figure 2k) [56, 57]. The coexistence of high‐valent metals and VO• sites afforded a synergistic catalytic surface, with electrophilic Mn^+^ centers accelerating OH^−^ adsorption and EG dehydrogenation and VO• sites lowering adsorption barriers by offering highly polarizable hydroxyl‐binding sites and promoting fast electron transfer and redox cycling. Collectively, these attributes provided HEOS with an enriched redox landscape and superior reaction kinetics, underpinning its enhanced catalytic performance for the EGOR and other alcohol oxidation reactions compared with that of HEO. Interestingly, HEOS featured F^+^ centers (singly ionized vacancies, VO•) whereas HEO featured F centers (neutral O vacancies, VO) and F^2+^ centers (doubly ionized vacancies, VO••) [58, 59, 60]. The abundant O vacancies in HEO favored lattice O–mediated (LOM) and adsorbed O–mediated (AOM) hydrogen atom transfer (HAT) along with mass transfer, thereby enabling formate generation through C—C bond cleavage and resulting in a catalytic efficiency and stability lower than those of HEOS [61, 62, 63]. The S contained in HEOS stabilized F^+^ centers with unpaired electrons, whereas the O vacancies in HEO existed in EPR‐silent charge states because of stronger lattice polarization, charge compensation, and magnetic interactions.

The O 1*s* spectra of HEO and HEOS (Figure S9) were deconvoluted into the peaks of lattice O (O^2−^), surface hydroxyl groups (OH^−^), and adsorbed water (H_2_O). For HEO, the O^2−^ peak (red) was more intense, indicating a higher proportion of stable lattice O, whereas the OH^−^ (green) and H_2_O (blue) components were also present in substantial amounts [3, 37, 40, 52]. In contrast, the spectrum of HEOS showed a markedly weaker O^2−^ signal and stronger OH^−^ and H_2_O signals, with OH^−^ being the dominant species. This trend suggests that S incorporation led to the partial replacement of lattice O^2−^ by S^2−^ and promoted the generation of O vacancies, which stabilized surface hydroxyl groups and enhanced surface reactivity (Figure S10) [64, 65]. The higher OH^−^ content of HEOS was particularly beneficial for EGOX, as the surface hydroxyls acted as active sites for AOM pathways and facilitated LOM pathways by providing labile O species favoring C—C bond cleavage and the oxidation of EG to formic acid [62, 63]. The lower proportion of strongly bound lattice O^2−^ in HEOS made its surface more flexible for redox reactions and minimized site blockage by rigid lattice O. Furthermore, the increased OH^−^/O^2−^ ratio of HEOS, together with its reduced H_2_O adsorption tendency, indicated a more catalytically active and less water‐poisoned surface. Therefore, the O vacancy–rich OH^–^ dominated surface of HEOS enabled more efficient EG activation and oxidation through coupled AOM and LOM mechanisms, as well as faster HAT, leading to superior catalytic performance for selective formic acid production.

### 2.2 Electrocatalytic Performance

**FIGURE 3:**
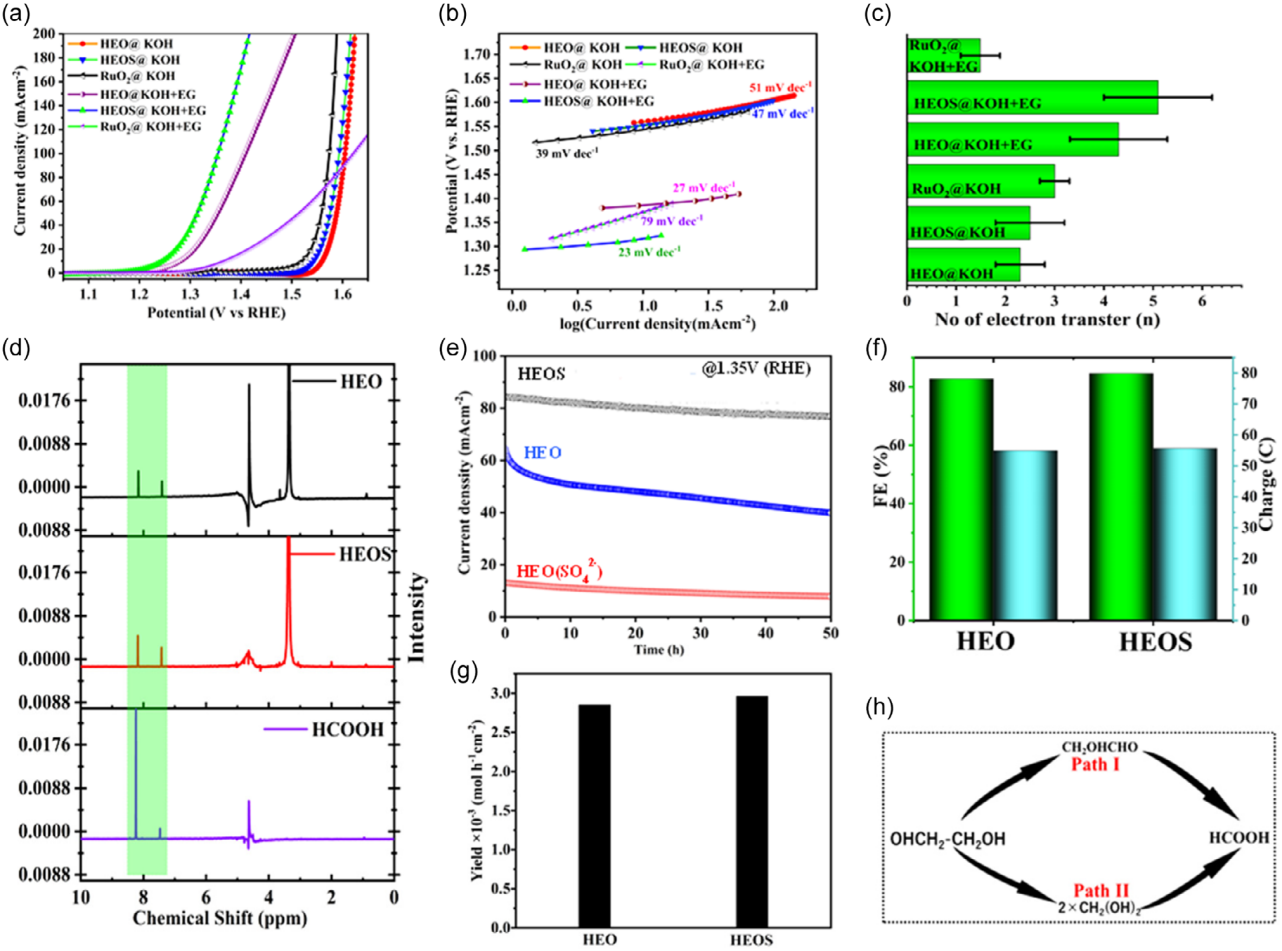
(a) Polarization curves of various electrocatalysts tested in different electrolytes and (b) corresponding Tafel plots. (c) Electron transfer numbers of different catalysts in various electrolytes derived using Tafel slope analysis. (d) 1H NMR spectra of postelectrolysis samples. (e) Results of chronoamperometric stability testing at a constant potential of 1.35 V versus the reversible hydrogen electrode. (f) Faradaic efficiency and cumulative charge passed (g) formic acid yield after 5.5 h of cyclic voltammetry measurements. (h) Mechanistic pathways proposed for selective ethylene glycol (EG) oxidation to formic acid.

HEOS outperformed HEO as an EGOR electrocatalyst (Figure 3). The corresponding polarization curves (Figure 3a) showed that HEOS achieved markedly higher current densities and lower onset potentials in 1 M KOH and 1 M KOH + 1 M EG, thus outperforming pristine and sulfate‐modified HEO (HEO(SO_4_^2−^); Figure S11 and Table S6). This conclusion was corroborated by the reduced Tafel slope of HEOS (23 mV dec^−1^, Figure 3b), which indicated faster reaction kinetics. The number of electrons transferred during oxidation (Figure 3c) for HEOS (5.1) closely approaches the theoretical value for complete EG conversion to formic acid and exceeded that obtained for HEO (4.3). The results of chronoamperometric stability testing at 1.35 V versus RHE (Figure 3e) revealed the long‐term operational durability of HEOS and showed that it surpassed those of HEO and HEO(SO_4_^2−^). This trend aligned with the mixing entropy values, with increased entropy correlating with the enhanced structural and electrochemical stability of HEOS. Interestingly, HEO(SO_4_^2−^) displayed a low catalytic activity and stability because of the formation of metal sulfate moieties during synthesis, as revealed by its S 2*p* spectrum (Figure S12).

This outcome was ascribed to the generation of catalytically inactive metal oxysulfates due to the combined use of tetramethylammonium hydroxide (TMOH) and Na_2_S. Thus, separate use of TMOH and Na_2_S is recommended for the efficient room‐temperature synthesis of HEO and HEOS with favorable EGOR performances. Postreaction ^1^H (Figure 3d) and ^13^C (Figures S13–S15) NMR spectroscopic analysis confirmed the formation of formic acid as the sole product, substantiating the high selectivity of HEOS. The ^1^H NMR signal at *δ* = 8.16 ppm observed after electrooxidation in 1 M KOH + 1 M EG closely matched the peak of pure formic acid (*δ* = 8.19 ppm), confirming that both HEOS and HEO selectively catalyzed the conversion of EG into formate. This conclusion was corroborated by ^13^C NMR spectroscopy, which revealed a carbonyl resonance matching that of formic acid and thereby confirmed the formation of formate as the sole detectable oxidation product.

The absence of ^1^H NMR (Figures S16 and S17) and ^13^C NMR (Figures S18 and S19) peaks corresponding to formaldehyde or other aldehydes underscores the highly selective formation of formic acid over HEOS and HEO. Moreover, no intermediates such as glycolaldehyde, glycolic acid, and glyoxalic acid were detected by NMR spectroscopy. The results of integrated NMR studies (Figures S20–S22) on posttreated samples after 100 cyclic voltammetry cycles and cumulative charge analysis (Figure S23) confirmed the efficiency of the examined catalysts. Comparative electrocatalytic performance analysis revealed that HEOS exhibited a higher faradaic efficiency (84.6%), accumulated charge (55.65 C), and formic acid production rate (2.9 × 10^−3^ mol h^−1^ cm^−2^) than HEO (82.7%, 54.91 C, and 2.85 × 10^−3^ mol h^−1^ cm^−2^, respectively) (Figure 3f,g), demonstrating a performance comparable with or superior to those of previously reported EGOR electrocatalysts (Table S6). The results of Tafel slope analysis suggested that EG oxidation over HEO and HEOS involved six‐electron transfer and the formation of a gem‐diol intermediate (Path II in Figure 3h). However, HEOS demonstrated a greater activity and selectivity for formic acid production because of the synergistic effect of S substitution and O vacancy stabilization. The S‐supported O vacancies in HEOS increased active site density and facilitated EG adsorption and activation, while the higher oxidation states of transition metals in this catalyst stabilized reactive O species (Figure S9), facilitating HAT. Valence‐band XPS analysis showed that the electronic structure of HEOS was markedly different from that of HEO (Figure S25), revealing a pronounced shift of the valence‐band maximum toward the Fermi level (2.1 eV for HEOS vs. 2.4 eV for HEO). This shift indicated a higher density of surface states and enhanced metallic character due to S incorporation and reflected the formation of covalent metal–S bonds and stabilized O vacancies, which enhanced charge transfer kinetics. This unique surface environment promoted the AOM and LOM pathways, enabling efficient C—C bond cleavage [4, 61, 62]. The increased density of states near the Fermi level facilitated the adsorption of hydroxyl intermediates, promoting LOM and AOM pathways during EGOX. The narrower band gap and electronic enrichment of HEOS were directly correlated with its superior catalytic performance, enabling efficient C—C bond cleavage and selective formate production. These findings validate the tailored electronic structure achieved through the synergy between S incorporation and O vacancies [65]. The abovementioned mechanisms collectively favored AOM and ensured complete EGOX to formic acid with minimal byproduct formation. S incorporation not only improved the intrinsic activity and selectivity but also enhanced the overall reaction kinetics and catalyst durability. Importantly, S contributed to O vacancy stabilization in HEOS by modifying the local electronic structure, inducing lattice strain, and promoting defect formation [58, 61, 62, 63, 64]. These effects reduced the vacancy formation energy and accelerated charge transfer while increasing the electrochemical active surface area of HEOS, thereby enhancing its catalytic performance (Figure S26 and Table S7).

Nyquist plots recorded at applied potentials from 1.3 to 1.6 V (Figure S24a,c) showed that HEOS exhibited markedly smaller semicircle diameters than HEO, indicating a lower charge transfer resistance (*R*_ct_) at the electrode‐electrolyte interface in the former case. This reduction in *R*_ct_ was ascribed to the synergistic effects of S substitution and O vacancies, which increased the density of active sites and enhanced electronic conductivity by facilitating charge delocalization through the lattice. Bode phase angle plots (Figure S24b,d) confirmed the superior kinetics of HEOS, revealing higher phase angles at intermediate frequencies and thus indicating improved charge transfer and capacitive behavior.

**FIGURE 4:**
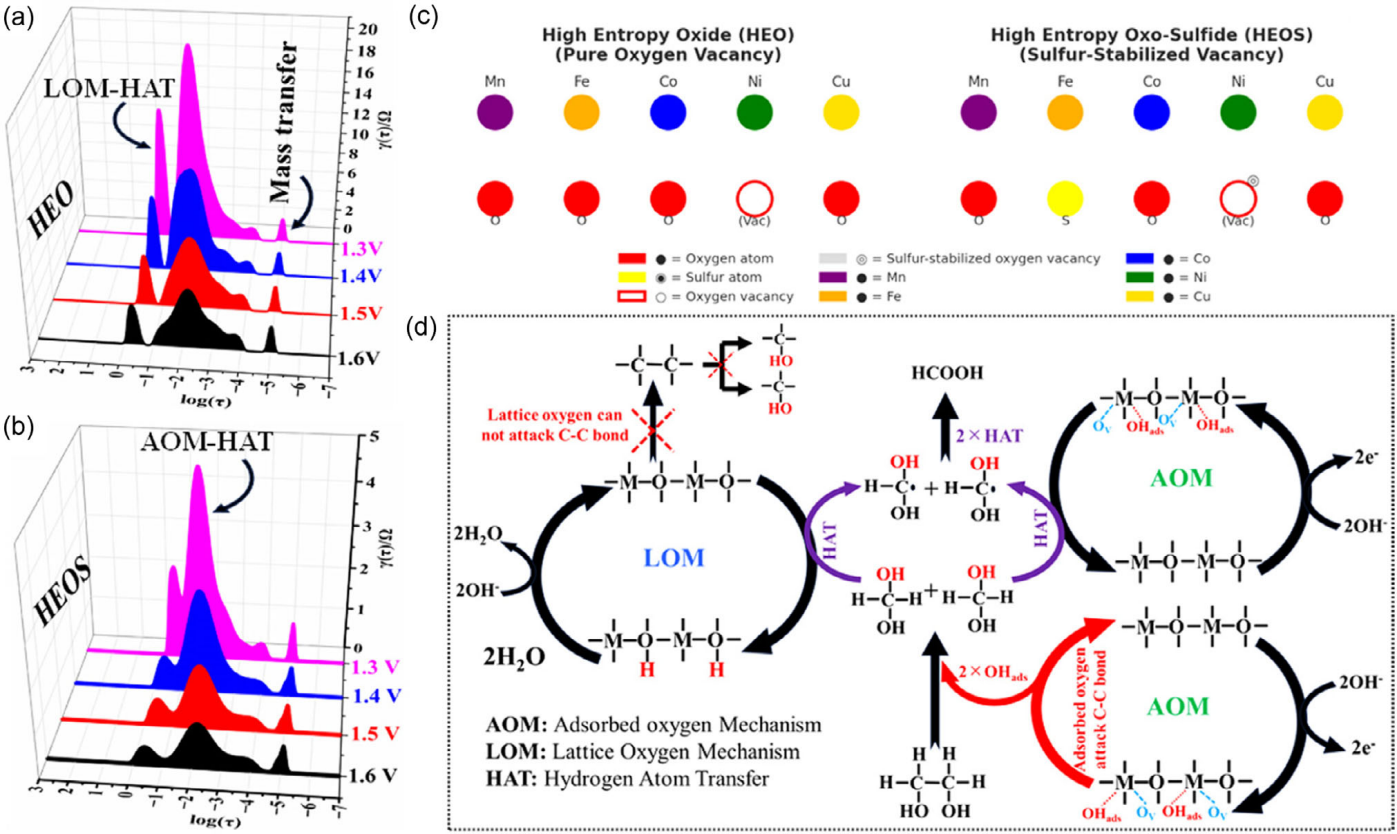
Distribution of relaxation time plots comparing charge transfer dynamics in (a) HEO and (b) HEOS. (c) Schematic of the crystal lattices of HEO and HEOS depicting the spatial distribution of O vacancies and their stabilization by S. (d) Proposed mechanism of S‐enabled and O vacancy‐driven EG oxidation illustrating the roles of lattice O‐mediated, adsorbed O‐mediated, and HAT processes in selective formic acid formation.

To gain mechanistic insights and decouple overlapping processes, we performed distribution of relaxation times (DRT) analysis, which resolves processes with similar time constants that would otherwise merge into a single feature in Nyquist or Bode plots enabling the separation of individual physical and chemical steps [2]. The results revealed that HEOS exhibited faster charge transfer kinetics, enhanced HAT rates, and higher LOM activity than HEO (Figure 4a,b). These observations indicated that S‐stabilized O vacancies in HEOS lowered the energy barrier for EG adsorption and oxidation, accelerating the coupled AOM and LOM pathways. The reaction probably proceeded via an unstable gem‐diol intermediate formed by the nucleophilic hydration of EG activated by OH_ads_ species at defect‐rich sites [4, 61, 62, 63, 64]. Mechanistically, the oxidation of EG to formic acid on HEOS involved the synergistic interplay of multivalent transition metal centers (Mn^n+^, Fe^n+^, Co^n+^, Ni^n+^, and Cu^n+^) and S‐stabilized O vacancies (Figure 4c). Mixed‐valence metal sites (Mn+/M^(n+δ)+^) dynamically generated electrophilic M—O and M—OH_ads_ species under anodic polarization. Coordinatively unsaturated metal sites bound OH_ads_, which facilitated EG activation through HAT mediated by lattice O and S vacancy–stabilized metal sites. In brief, concerted AOM facilitated the nucleophilic hydration of the adsorbed EG, yielding an unstable gem‐diol intermediate (HO—CH_2_—OH) through OH_ads_ transfer. This intermediate underwent sequential dehydrogenation via HAT to afford a gem‐diol radical ((HO)_2_—CH^•^), which was further oxidized at M^(n+δ)+^ sites to selectively produce formic acid and no detectable byproducts (Figure 4d). Moreover, the mixed‐valence centers in HEOS provided a flexible redox environment, optimized intermediate binding energies, and enhanced charge transfer kinetics, increasing the selectivity for formic acid while suppressing overoxidation to CO_2_. The S induce O vacancy engineering further improved selectivity and prevented electrode passivation by stabilizing O vacancies generated during pre‐electrooxidation‐induced reconstruction. In contrast, HEO lacked S stabilization and relied predominantly on M—O bond‐based LOM and HAT pathways, which were less effective at C—C bond cleavage and led to a lower faradaic efficiency. Thus, O vacancies alone were catalytically less active without an appropriate surface chemistry. These results underscore the critical role of S‐stabilized O vacancies in HEOS in enabling efficient C—C bond cleavage, sustained activity, and high selectivity for formic acid, establishing HEOS as a highly promising catalyst for controlled polyol electrooxidation [62].

**FIGURE 5:**
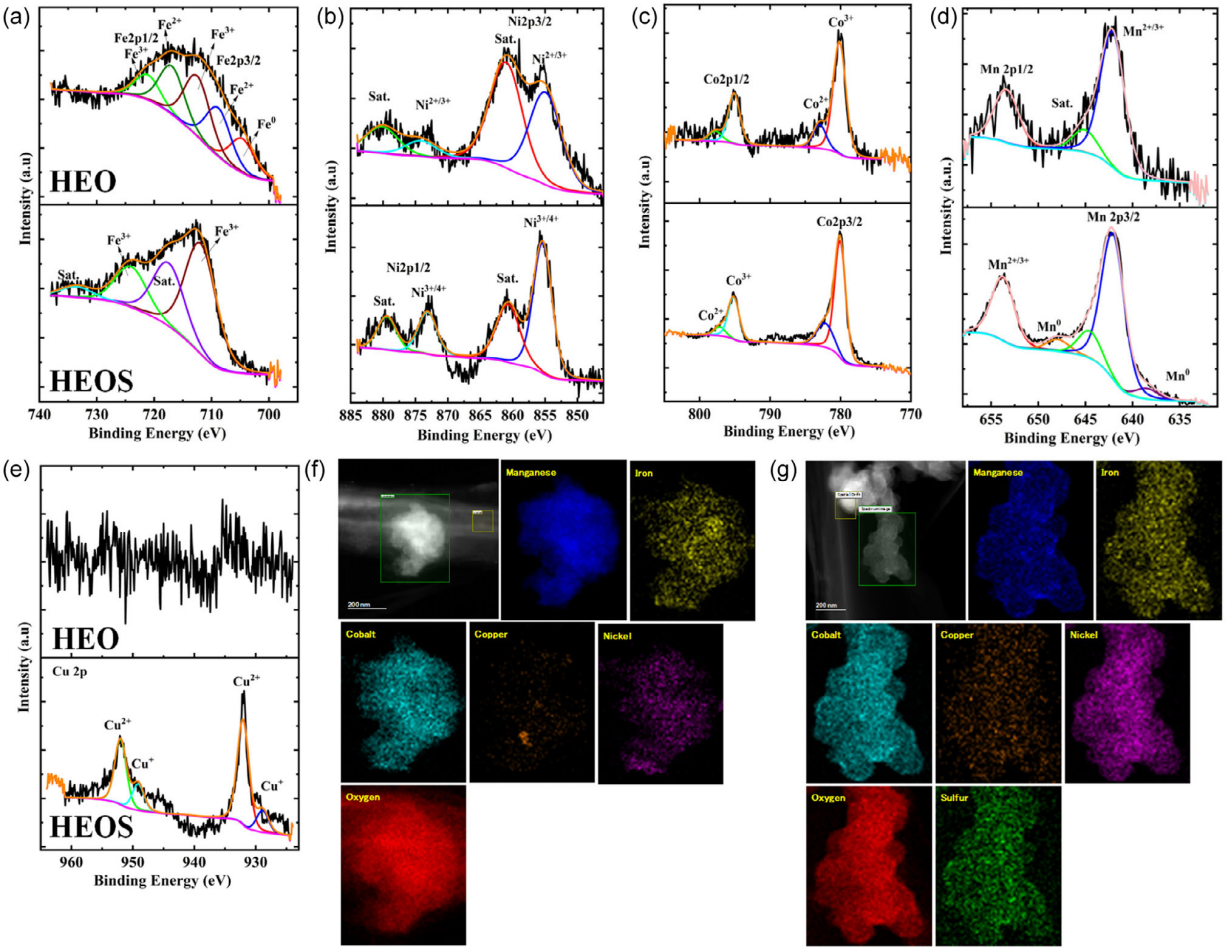
Deconvoluted (a) Fe 2*p*, (b) Ni 2*p*, (c) Co 2*p*, (d) Mn 2*p*, and (e) Cu 2*p* spectra of post treated HEOS and HEO. STEM‐EDX elemental mappings of post treated (f) HEO and (g) HEOS.

The posttreated characterization of HEOS and HEO revealed the superior catalytic activity and stability of the former, underpinned by the surface redox behaviors and electronic structures of the active elements (Figure 5a–e). The Fe 2*p* spectrum of HEO showed that O vacancies and lattice disorder stabilized a mixed Fe^2+^/Fe^3+^ state, suppressing the Fe^3+^ shake‐up satellites because of enhanced screening. In contrast, the Fe 2*p* spectrum of HEOS was dominated by the peaks of Fe^3+^ with clearly resolved shake‐up satellites, which was attributed to the S‐induced oxidation of Fe^2+^ and enhanced electron localization (Figure 5a) [66, 67]. Similarly, the corresponding Mn 2*p* spectra indicated that HEO maintained a stable mixed Mn^2+^/Mn^3+^ state with characteristic Mn^3+^ satellites, whereas HEOS featured metallic Mn^0^ alongside Mn^2+^/Mn^3+^ and stronger satellites. These findings reflected the more reducing surface conditions and high‐defect weakly bonded sulfide environment of HEOS, which destabilized higher oxidation states under the reaction conditions (Figure 5d) [68]. The related Cu 2*p* spectra showed that in HEOS, Cu^+^ (3*d* ^10^) was oxidized to Cu^2+^ (3*d* ^9^) during EGOX to compensate for charge transfer to adsorbed intermediates, with the Cu^2+^ component being dominant and shake‐up satellites suppressed because of the highly covalent Cu—S bonding and charge delocalization (Figure 5e) [68, 69]. The Co 2*p* spectra of HEO and HEOS showed suppressed shake‐up satellites, as the oxidation of Co^2+^ to Co^3+^ favored more covalent and delocalized electronic states, diminishing satellite intensity (Figure 5c) [66, 67].

Ni 2*p* spectra revealed the presence of mixed Ni^2+^/Ni^3+^ states in both materials, with HEOS exhibiting a higher Ni^2+^/Ni^3+^ ratio and minimally altered satellite intensity, consistent with the S‐induced charge redistribution and stabilization of higher oxidation states, whereas HEO retained more Ni^2+^ because of O vacancy stabilization (Figure 5b) [66, 67, 68]. Collectively, the deconvoluted XPS spectra (Figure 5a–e) demonstrated that HEOS featured key active elements (Fe^3+^, Ni^3+^, Cu^2+^, Co^3+^, and Mn^3+^) in higher oxidation states than HEO because of the O vacancies and lattice disorder in the latter material [70].

STEM‐EDX analysis (Figure 5f,g) demonstrated that the homogeneous elemental distribution and structural integrity of HEOS were preserved after prolonged EGOX, underscoring the superior durability of this material. In contrast, HEO exhibited signs of Cu migration and agglomeration following EGOX, as well as surface Cu loss. This behavior was ascribed to the O‐rich vacancy‐defective lattice of HEO, which facilitated the partial reduction of Cu^2+^ to metallic Cu^0^ and subsequent clustering, thereby decreasing the number of active Cu sites [3]. In HEOS, S substitution stabilized Cu^2+^ via covalent Cu—S bonding and defect‐induced charge redistribution, suppressing Cu reduction and agglomeration and helping maintain a uniform Cu distribution. These structural and compositional observations corroborate the mechanistic insights provided by DRT analysis, which revealed that S incorporation enhanced the AOM and LOM pathways while accelerating HAT. Collectively, these data highlight the critical role of S substitution in modulating the electronic structure and defect chemistry, stabilizing high‐valence active sites, and facilitating synergistic AOM, LOM, and HAT pathways [4, 61, 62]. This unique combination of features underpins the superior EGOX performance and stability of HEOS and make it an effective and durable catalyst for selective polyol electrooxidation.

### 2.3 Reaction Mechanism

To further validate the proposed reaction mechanism involving LOM and AOM pathways together with accelerated HAT, EGOX was conducted in an H_2_^18^O‐labeled alkaline electrolyte, and electrolyte samples were collected after 4, 6, and 10 h of chronoamperometry at a constant potential. ^1^H NMR analysis confirms that formate remains the sole detectable liquid product throughout the reaction, demonstrating that isotopic labeling does not alter product selectivity (Figure S27). Importantly, NMR further reveals that oxygen atoms in the formate product originate from both ethylene glycol and the aqueous electrolyte, consistent with the dual‐oxygen participation mechanism illustrated in Figure 4d. Ex situ FTIR spectroscopy provides direct vibrational evidence supporting this conclusion. After reaction in the unlabeled electrolyte, HEOS exhibits strong carboxylate‐related bands at ≈1428 and ≈1328 cm^−1^, assigned to the asymmetric and symmetric stretching modes of formate (v_as_(COO^−^) and v_s_(COO^−^)), respectively, together with accompanying C—O stretching features at ≈1095 and ≈1018 cm^−1^ (Figure S28). Upon performing EGOX in H_2_^18^O, this carboxylate features undergo pronounced reorganization and red‐shifting, with the v(COO^−^) envelope evolving into bands at ≈1532, ≈1479, and a dominant feature at ≈1389 cm^−1^, alongside shifts in the C—O region to ≈1127–1096 and ≈974 cm^−1^ (Figure S28). These isotope‐sensitive changes provide clear evidence for electrolyte‐derived oxygen incorporation into the formate moiety via the AOM pathway, mediated by surface M–O^18^H species, analogous to oxygen exchange and adsorbate‐derived oxygen participation observed on Ir‐oxide OER catalysts using ^18^O labeling [71]. Concurrently, the lattice vibrational region shows systematic evolution: metal–oxygen framework modes initially observed in the ≈520–800 cm^−1^ range before reaction progressively shift and redistribute after electrolysis, consistent with partial replacement of M–^16^O by heavier M–^18^O species and confirming active lattice oxygen exchange through the LOM pathway, in line with lattice oxygen exchange and participation documented for IrO_2_‐based systems [72]. Notably, this lattice‐level isotope exchange is rapid and pronounced in HEOS, reflecting the high density of sulfur‐stabilized oxygen vacancies that facilitate oxygen mobility and replenishment. At early reaction stages, broad hydroxyl stretching features in the 3200–3500 cm^−1^ region, together with transient C—O vibrations in the ≈1100 cm^−1^ range, are observed, indicating nucleophilic hydration of adsorbed ethylene glycol to form a short‐lived gem‐diol‐like intermediate (Figure S28). The rapid disappearance of these hydroxyl and C—O features, combined with the absence of aldehyde‐related bands in the 1700–1750 cm^−1^ region in both FTIR and NMR spectra, confirms fast HAT steps following gem‐diol formation, consistent with gem‐diol‐mediated oxidation and hydride/H‐atom transfer pathways resolved by in situ vibrational spectroscopy and isotope tracing on oxide catalysts [73]. These observations support a concerted oxidation mechanism in which sulfur‐stabilized oxygen vacancies and high‐valence metal centers in HEOS promote sequential HAT from the gem‐diol intermediate, leading directly to C—C bond cleavage and selective formate formation. Collectively, the combined ^18^O isotopic labeling, FTIR vibrational analysis, and NMR product identification provide compelling experimental evidence that EGOX over HEOS proceeds via a gem‐diol‐mediated pathway governed by synergistic AOM and LOM oxygen participation and accelerated HAT kinetics, accounting for its superior activity, selectivity, and durability relative to HEO.

### 2.4 PET Degradation

To investigate the practical viability of HEOS as an EGOR electrocatalyst, we used it to upcycle PET waste into formic acid and H_2_ via alkaline hydrolysis followed by electrochemical oxidation (Figure S29). The hydrolysis of PET yielded PTA and EG, as confirmed by ^1^H NMR spectroscopy (Figures S30 and S31). The sharp resonance at *δ* = 3.4 ppm verified the presence of EG (Figures S30 and S32), with its concentration estimated as ≈1 M.

**FIGURE 6:**
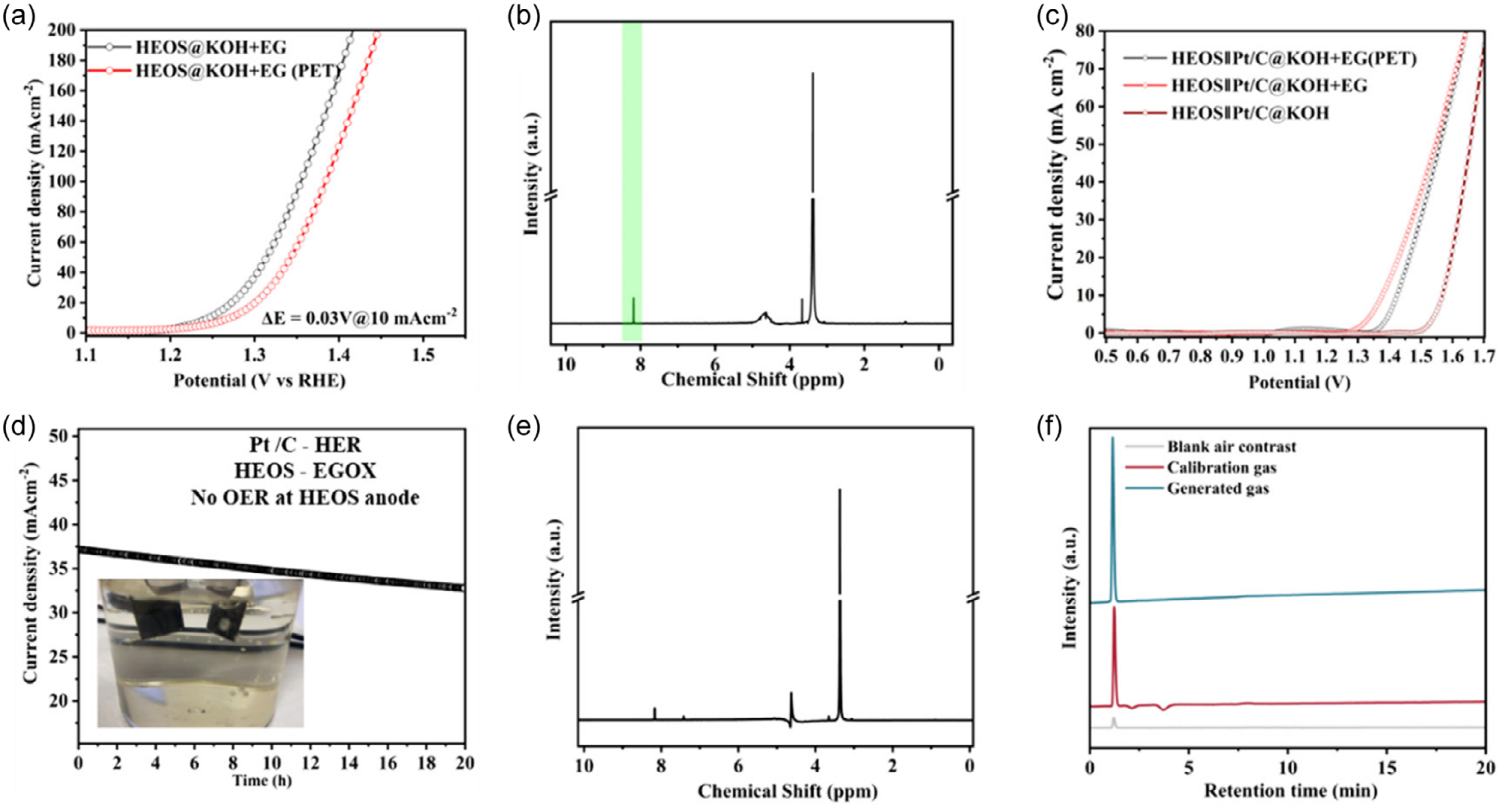
(a) Linear sweep polarization curves of HEOS in 1 M KOH recorded using reagent‐grade and polyethylene terephthalate (PET)‐derived EG as anodic substrates. (b) 1H NMR spectrum of the product of PET‐derived EG electrooxidation obtained in the two‐electrode configuration. (c) Two‐electrode cell polarization plots recorded for HEOS as the anode material and commercial Pt/C as the cathode material in three electrolyte systems, namely 1 M KOH, 1 M KOH + EG, and 1 M KOH + PET‐derived EG. (d) Chronoamperometric response over 20 h of operation for PET‐derived EG. (e) 1H NMR spectrum of the reaction product after 20 h of chronoamperometry testing. (f) Gas chromatographic profiles of cathodic samples.

The PET‐derived EG solution was utilized as an electrolyte in a two‐electrode electrolyzer with a Pt/C cathode for the H_2_ evolution reaction (HER) and HEOS anode for the EGOR. The onset potential observed for PET‐derived EG marginally (by 0.03 V) exceeded that observed for reagent‐grade EG, which revealed the compatibility of HEOS with real‐life feedstocks (Figure 6a). Linear sweep voltammetry tests revealed an increase in the current density and decrease in the anodic onset potential with the increasing EG concentration (Figure S33). Electrochemical tests were conducted using three electrolytes, namely (i) 1 M KOH (OER benchmark), (ii) 1 M KOH + 1 M EG, and (iii) 1 M KOH + PET‐derived EG, in a two‐electrode set‐up with HEOS as the anode material and Pt/C as the cathode material (Figure 6c). The respective cell voltages at 10 mA cm^−2^ were 1.57, 1.37, and 1.42 V, revealing substantial energy savings due to the substitution of the OER for the EGOR (Figure 6c) [74].

The electrochemical response of the PET‐derived hydrolysate closely paralleled that of reagent‐grade EG, which suggested negligible interference from coexisting species. The ^1^H NMR spectrum of the product of PET‐based EG oxidation featured a signal at *δ* = 8.19 ppm, indicating selective formate formation (Figures 6b and S34). Taking advantage of the highly selective formate production during the HEOS‐catalyzed electrooxidation of PET‐derived EG and the low onset potential of this process, we constructed an EGOR/HER coupled system in an H‐type cell, with the EGOR and HER serving as the anodic and cathodic reactions, respectively (EGOX||HER). HEOS and commercial Pt/C were employed as the anode and cathode active materials, respectively. The cell voltage at a current density of 10 mA cm^−2^ (1.39 V) was lower than that of the traditional OER/HER system, suggesting the higher electrocatalytic efficiency and lower energy consumption of our EGOX||HER system (Figure S35 and Table S8). Chronoamperometric testing conducted at 1.5 V in an H‐type cell demonstrated that HEOS retained 92% of its initial activity (≈39 mA cm^−2^) after 20 h of continuous operation, highlighting its excellent durability and potential for practical application (Figure 6d). The stability of HEOS during EGOX is further corroborated by the posttreated S 2*p* XPS spectra, which confirm the preservation of sulfur chemical states after prolonged electrochemical operation (Figure S36). Based on the results of ^1^H NMR spectroscopy analysis, formate was identified as the exclusive oxidation product formed at the anode, with the corresponding faradaic efficiency determined as 57.84% (Figures 6e and S37). Gas chromatography analysis confirmed H_2_ evolution at the cathode without any detectable side products (Figure 6f). These results demonstrate that HEOS enables the efficient coupling of EG‐derived plastic upcycling with sustainable H_2_ production, underscoring its potential as a dual‐functional catalyst for renewable energy and circular chemical manufacturing.

## 3 Experimental Section

### 3.1 HEOS and HEO Synthesis

MnCl_2_ · 4H_2_O, FeCl_2_·4H_2_O, CoCl_2_ · 6H_2_O, NiCl_2_ · 6H_2_O, and CuCl_2_·2H_2_O were dissolved in water (0.05 M each) under ambient conditions (≈298 K), and the resulting solution (10 mL) was treated with aqueous Na_2_S (20 mL, 1 M). The resulting black suspension was vigorously stirred at room temperature for 3 h, and the precipitate was washed several times with a 1:1 (v/v) mixture of distilled water and ethanol and air‐dried at room temperature. HEO was synthesized using the same metal salts as described elsewhere [3]. The simultaneous presence of TMOH and Na_2_S during synthesis led to the formation of an electrocatalytically unstable high‐entropy oxysulfate denoted as HEO(SO_4_^2−^).

### 3.2 Synthesis of PET‐Derived EG

Waste PET (4 g) was dissolved in aqueous KOH (100 mL; 4 M) at 80°C under constant stirring. After complete dissolution, deionized water (400 mL) was added to the mixture, and stirring was continued for 30 min. The solution was dropwise supplemented with 4 M H_2_SO_4_. The PTA precipitate was removed, and the EG‐rich supernatant was collected by centrifugation at 5000 rpm (10000 gravity) for 10 min (Figure S29).

## 4 Conclusion

A S‐induced oxygen‐vacancy–activated electrocatalyst (HEOS) was synthesized via a single‐step, room‐temperature sulfur incorporation into HEO, exhibiting high Faradaic efficiency and excellent selectivity toward ethylene glycol electrooxidation and water electrolysis. S incorporation stabilized O vacancies through covalent M‐S bonding and defect‐induced charge redistribution while reinforcing structural integrity under oxidative conditions. HEOS outperformed HEO in terms of catalytic activity and selectivity for C—C bond cleavage and formic acid production, which was ascribed to the optimized electronic structure of the former and synergistic effects of S incorporation and vacancy engineering. S atoms stabilized high‐valent metal states via covalent metal‐S interactions, enhanced defect chemistry, and promoted the adsorption of hydroxyl species and lattice O activation, thus enabling precise control over C—C bond cleavage pathways. Mechanistic DRT analysis revealed that S incorporation enhanced both LOM and AOM pathways while accelerating HAT therby facilitated the conversion of EG into valuable chemicals. Notably, substituting the anodic OER with the EGOR markedly reduced the water electrolysis overpotential, enabling sustainable and energy‐efficient H_2_ production coupled with plastic waste valorization. This work introduces a robust and scalable synthetic route to HEOSs with tailored defect profiles and establishes their utility as multifunctional electrocatalysts for decarbonized H_2_ generation and plastic‐to‐chemical conversion. The obtained insights pave the way for the rational design of next‐generation electrocatalysts to address pressing challenges in the energy transition and circular chemical manufacturing.

## Supporting Information

Additional supporting information can be found online in the Supporting Information section. **Supporting Fig. S1:** Selected area electron diffraction (SAED) pattern and energy‐dispersive X‐ray spectroscopy (EDS) profile of HEOS. The SAED pattern displays diffraction rings indicative of polycrystalline domains and successful phase formation. The EDS profile reveals the uniform distribution of constituent transition metals and S, supporting compositional homogeneity and successful S incorporation into the HEO framework. **Supporting Fig. S2:** SAED pattern and EDS profile of HEO. The SAED pattern reveals a polycrystalline nature and features well‐defined diffraction rings indicative of a multiphase cubic structure. The EDS profile reveals the homogeneous distribution of constituent transition metals, indicating compositional uniformity and successful element integration into the HEO matrix. **Supporting Fig. S3:** X‐ray diffraction patterns of HEOS and HEO confirming phase formation and crystallinity. The diffraction peaks correspond to a multiphase cubic structure, with comparable lattice parameters observed for both materials. The absence of impurity phases indicates that S was incorporated into the HEO lattice without altering the overall phase symmetry. The HEOS sample exhibits significantly enhanced diffraction intensity with prominent broad peaks at approximately 20° and 35° 2*θ*, indicating structural modification upon sulfur incorporation. The diffraction patterns demonstrate distinct differences in crystallinity and phase composition between the two materials. **Supporting Fig. S4:** Elemental composition of HEOS determined by STEM–EDX analysis. The data confirm the homogeneous incorporation of transition metals and S and support the configurational uniformity and successful synthesis of the high‐entropy phase. **Supporting Fig. S5:** Elemental composition of HEO determined by STEM–EDX analysis. The data confirm the uniform elemental distribution and successful incorporation of all target metal species and O into the high‐entropy matrix, supporting the synthesis of a compositionally stable HEO framework. **Supporting Fig. S6:** High‐resolution transmission electron microscopy images of (a) HEO and (b) HEOS revealing lattice fringes indicative of high crystallinity in both cases. HEOS displays an enhanced defect density and minimally distorted lattice structures, consistent with S incorporation and O vacancy formation, whereas HEO exhibits a more uniform lattice alignment. These structural features support the important role of defect engineering in modulating catalytic activity. **Supporting Fig. S7:** Raman spectra of HEOS and HEO, representing the presence of M—S and M—O bond. **Supporting Fig. S8:** X‐ray photoelectron survey spectra of HEO (bottom) and HEOS (top). The spectra confirm the presence of transition metals and O in HEO and additional S in HEOS, indicating successful S incorporation into the high‐entropy matrix. **Supporting Fig. S9:** Deconvoluted O 1s X‐ray photoelectron spectra of HEO and HEOS. For HEO, the dominant peaks correspond to lattice O (O^2−^) and adsorbed hydroxyl groups. For HEOS, the relative intensity of O vacancy–related components increases because of S incorporation, suggesting enhanced lattice disorder and vacancy concentration. These spectral features corroborate the defect‐mediated electronic structure modulation and support the synergistic role of S–O vacancies in catalytic activity enhancement. **Supporting Fig. S10:** Deconvoluted S 2*p* X‐ray photoelectron spectrum of HEOS (Residual STD 0.6396). The spectrum reveals S 2*p*_3/2_ and S 2*p*_1/2_ doublets, indicating the presence of both lattice incorporated sulfide species (S^2−^) and surface‐bound S moieties. The dominant low‐binding energy component reflects strong covalent metal–S bonding, while higher‐energy contributions suggest oxidized S species or partial sulfate formation. These features confirm S integration and support its role in electronic structure modulation and vacancy stabilization within the HEOS lattice. **Supporting Fig. S11:** Linear sweep voltammograms of different EG oxidation electrocatalysts recorded in 1 M KOH. The curves reveal distinct onset potentials and current densities, with HEOS exhibiting a lower onset potential and higher catalytic current and thus showing superior electrocatalytic activity for EG oxidation. Sulfate‐functionalized HEO (HEO(SO_4_^2−^)) shows relatively sluggish kinetics. **Supporting Fig. S12:** Deconvoluted S 2*p* X‐ray photoelectron spectrum of HEO(SO_4_^2−^) showing characteristic S 2*p*_3/2_ and S 2p_1/2_ doublets at higher binding energies, consistent with surface bound sulfate. These peaks distinguish oxidized S states from lattice‐incorporated sulfide (S^2−^) and confirm the presence of terminal sulfate groups. The absence of low‐binding‐energy components suggests the minimal incorporation of reduced S species, supporting the chemical nature of surface modification via sulfate functionalization. **Supporting Fig. S13:** ^13^C NMR spectra of the products of HEOS‐catalyzed EG electrooxidation and reference formic acid in 1 M aqueous KOH. The former spectrum shows a resonance matching that of the formic acid reference. The absence of additional peaks confirms selective formate formation under alkaline conditions. The inclusion of the internal standard enables the quantitative comparison of product concentrations and spectral assignment validation. **Supporting Fig. S14:** Integrated ^13^C NMR spectrum of pure formic acid in 1 M aqueous KOH. **Supporting Fig. S15:** Integrated ^13^C NMR spectrum of the product of HEOS‐catalyzed EG electrooxidation. The spectrum displays a dominant signal corresponding to the carbonyl carbon of formate, confirming the selective conversion of EG to formic acid. **Supporting Fig. S16:** ^1^H NMR spectrum of formaldehyde recorded in 1 M KOH + 1 M EG using CHCl_3_ as an internal standard. The spectrum displays characteristic formaldehyde peaks, enabling comparative analysis across different alkaline media. Signal shifts and relative intensities reflect the chemical environment and reactivity of formaldehyde under alkaline conditions, with CHCl_3_ providing a stable reference peak for quantitative calibration. **Supporting Fig. S17:** Integrated ^1^H NMR spectrum of formaldehyde recorded in 1 M KOH using CHCl_3_ as an internal standard. **Supporting Fig. S18:** ^13^C NMR spectrum of pure formaldehyde recorded in 1 M aqueous KOH. **Supporting Fig. S19:** Integrated ^13^C NMR spectrum of pure formaldehyde recorded in 1 M aqueous KOH. **Supporting Fig. S20:** Integrated ^1^H NMR spectrum of formic acid recorded in 1 M KOH using CHCl_3_ as an internal standard. **Supporting Fig. S21:** Integrated ^1^H NMR spectrum of the product of HEOS‐catalyzed EG oxidation in 1 M KOH + 1 M EG recorded using CHCl_3_ as an internal standard. The spectrum features a singlet corresponding to the formyl proton of formic acid, confirming the selective conversion of EG to formate. The absence of additional peaks suggests minimal byproduct formation. **Supporting Fig. S22:** Integrated ^1^H NMR spectrum of the product of HEO‐catalyzed electrooxidation recorded in 1 M KOH + 1 M EG using CHCl_3_ as an internal standard. **Supporting Fig. S23:** Cyclic voltammograms recorded over 100 consecutive cycles and the corresponding acquired charge (*Q*) per cycle for HEOS and HEO electrodes. **Supporting Fig. S24:** (a,c) Nyquist and (b,d) Bode phase angle plots for (a,b) HEO and (c,d) HEOS. **Supporting Fig. S25:** Valence band X‐ray photoelectron spectra of HEO and HEOS. The spectra reveal differences in valence band edge positions, reflecting the electronic structure modulation induced by S incorporation. HEOS exhibits a shallower valence band maximum than HEO, which indicates an altered charge distribution and enhanced electronic conductivity. These shifts support the synergistic role of S and O vacancies in tailoring the density of states for improved electrocatalytic activity. **Supporting Fig. S26:** Cyclic voltammograms of (a) HEO and (c) HEOS recorded at varying scan rates and (b,d) corresponding linear fits of anodic/cathodic current–scan rate plots. **Supporting Fig. S27:** ^1^H NMR spectra of the electrolyte collected during ethylene glycol electrooxidation over HEOS after 4, 6, and 10 h of chronoamperometric operation. The dominant resonance at ≈8.15 ppm corresponds to formate, confirming its formation as the primary liquid product throughout the reaction, while the absence of additional proton signals indicates high product selectivity and stability over prolonged electrolysis. **Supporting Fig. S28:** Fourier‐transform infrared (FTIR) spectra of HEOS collected before electrochemical testing, after electrochemical testing, and after the isotopic labeling experiment. The comparison highlights the evolution of surface vibrational features upon electrochemical operation and confirms the structural integrity of HEOS following the H_2_^18^O‐assisted isotopic labeling test. **Supporting Fig. S29:** Schematic of polyethylene terephthalate (PET)‐derived EG recycling. **Supporting Fig. S30:** ^1^H NMR spectrum of a PET solution before (black) and after (red) alkaline hydrolysis in 1 M KOH. The initial PET solution shows characteristic polymer signals, whereas the solution after hydrolysis exhibits peaks at *δ* = 3.4 ppm and other shifts confirming the depolymerization of PET into EG and terephthalic acid as the main products. **Supporting Fig. S31:** ^1^H NMR spectrum of the PET solution in 1 M KOH featuring the signals of EG and terephthalic acid as the main products and thus confirming the depolymerization of PET. **Supporting Fig. S32:** ^1^H NMR spectrum of the PET solution after alkaline hydrolysis. The characteristic peak at *δ* = 3.4 ppm confirms the formation of EG. **Supporting Fig. S33:** Linear sweep voltammograms of HEOS recorded at varying concentrations of EG in 1 M KOH. **Supporting Fig. S34:** ^1^H NMR spectrum of the product obtained by the HEOS‐catalyzed oxidation of PET‐derived EG in 1 M KOH. **Supporting Fig. S35:** (a) Photograph of the H‐type two‐electrode setup used for coupled EG oxidation and H_2_ evolution, featuring HEOS as the anode material and Pt/C as the cathode material. (b) Polarization curves obtained under various electrolyte conditions, illustrating the electrocatalytic activity and onset potentials for EG oxidation and overall water splitting. **Supporting Fig. S36:** Deconvoluted S 2*p* X‐ray photoelectron spectrum of HEOS after electrochemical testing. **Supporting Fig. S37:** ^1^H NMR spectrum of the product obtained after 20 h of chronoamperometric testing in a two‐electrode setup (electrode surface area = 0.0625 cm^2^). CHCl_3_ was used as an internal standard to quantify the product concentration. **Supporting Table S1:** Elemental composition of the high‐entropy oxide (HEO) determined by scanning transmission electron microscopy coupled with energy‐dispersive X‐ray spectroscopy (STEM‐EDX). **Supporting Table S2:** Elemental composition of the high‐entropy oxysulfide (HEOS) determined by STEM‐EDX. **Supporting Table S3:** Melting points of the transition metals contained in HEO and HEOS. **Supporting Table S4:** Binary mixing enthalpies of transition metal alloys. **Supporting Table S5:** Thermodynamic parameters calculated for HEO and HEOS. **Supporting Table S6:** Performances of different catalysts for ethylene glycol (EG) oxidation in alkaline media. **Supporting Table S7:** Double layer capacitances (*C*_dl_) and electrochemically active surface areas (ECSAs) of HEOS and HEO. **Supporting Table S8:** Cell voltages of various electrochemical coupling systems.

## Conflicts of Interest

The authors declare no conflicts of interest.

## Supporting information

Supplementary Material
