# Supplementary material for: Sulfur‐Stabilized High Entropy Oxysulfides Enable Efficient C—C Bond Cleavage in Ethylene Glycol Electrooxidation for Sustainable Plastic Upcycling to Formate
Source: ChemSusChem. 2026 Mar 3;19(5):e202502529. doi: 10.1002/cssc.202502529 (PMC12954829; doi:10.1002/cssc.202502529)
Supplement: Supplementary file 1 — Supplementary Material [file CSSC-19-e202502529-s001.pdf]

Supporting Information

Sulfur-Stabilized High-Entropy Oxysulfides Enable Efficient C–C Bond Cleavage in Ethylene Glycol Electrooxidation for Sustainable Plastic Upcycling to Formate

Saikat Bolar [a], Akitaka Ito*[a], Chunyu Yuan[a], Meiyi Wang[b], Akira Yamaguchi[b], Masahiro Miyauchi[b], Takeshi Fujita*[a]

[a] School of Engineering Science, Kochi University of Technology, 185 Miyanokuchi, Tosayamada, Kami City, Kochi 782-8502, Japan

[b] Department of Materials Science and Engineering, School of Materials and Chemical Technology, Institute of Science Tokyo, Meguro, Tokyo, Japan

E-mail: ito.akitaka@kochi-tech.ac.jp, fujita.takeshi@kochi-tech.ac.jp

Experimental Section

Chemicals: $MnCl_2\cdot4H_2O$, $FeCl_2\cdot4H_2O$, $CoCl_2\cdot6H_2O$, $NiCl_2\cdot6H_2O$, $CuCl_2\cdot2H_2O$, Na_2S, ethanol, dimethylformamide, formic acid, formaldehyde, ethylene glycol (EG; AR, 98%), chloroform, Nafion™ solution (5.0 wt%), Pt/C (46.8 wt%), KOH, and H_2SO_4 were purchased from Wako Chemicals (Japan). RuO_2 and aqueous tetramethylammonium hydroxide were purchased from Sigma-Aldrich. Isopropyl alcohol was purchased from Kanto Chemical Co., Inc. ^{8}O-labeled water ($H_2{}^{18}O$, 97% ^{18}O enrichment; CAS RN®: 14314-42-2) was purchased from Cambridge Isotope Laboratories, Inc. and stored at room temperature. All chemicals were used as received without any further purification. PET waste was randomly collected from the KUT campus dustbin and cut into small pieces for further use.

Material Characterization: X-ray diffraction patterns were obtained using a Rigaku RINT 2000 diffractometer (JAPAN) equipped with a monochromatic Cu K_α radiation source (λ = 1.5406 Å) and operated at 40 kV and 40 mA. Transmission electron microscopy (TEM) imaging was performed using a JEM-ARM200F NEOARM microscope (JEOL) equipped with aberration correctors for image and probe-forming lens systems (CEOS GmbH). Energy-dispersive X-ray spectroscopy analysis was conducted using a JED-2300T (JEOL) detector. TEM and scanning TEM (STEM) observations were performed at an accelerating voltage of 200 kV. X-ray photoelectron spectra were acquired using an AXIS Ultra DLD system (Shimadzu) with a monochromatic Al K_α radiation source. ^{1}H and ^{13}C NMR spectra were recorded using a JEOL JNM-ECZ400S spectrometer operating at 400 MHz. $CHCl_3$ was used as an internal standard. $CHCl_3$ (3.95 µL) was added to each stock solution (1 mL) before measurements to afford a final internal-standard concentration of 50 mM. NMR spectra were recorded in regular water without the use of deuterated solvents. In the reference sample, the EG signal appeared at $\delta \approx 3.40$ ppm, with the $CHCl_3$ peak observed at $\delta \approx 7.46$ ppm. Despite the absence of deuterated solvents, the water signal could be appropriately suppressed. The integration of the EG peak relative to that

of the internal standard enabled the accurate quantification of the concentrations of EG and its oxidation products. Gas chromatographic analysis was performed on a calibrated gas chromatograph equipped with a thermal conductivity detector using high-purity Ar as the carrier gas. The analysis was conducted at ambient temperature using appropriate sampling intervals to quantify the evolved H_2 and assess the selectivity of the cathodic reaction. All measurements were carried out at room temperature using consistent acquisition parameters. Electron spin resonance (ESR) measurements were conducted at 110 K using a Bruker ESR Nano spectrometer in a nitrogen atmosphere under dark conditions. The sample was loaded into a 4 mm-diameter quartz tube, extending more than 4 mm from the bottom of the tube.

Raman spectra of HEO and HEOS were recorded using a micro-Raman spectrometer (Renishaw inVia Reflex) equipped with a 532 nm excitation laser (λ = 532.5 nm). To prevent laser-induced heating, structural damage, or unintended reduction of the samples, the laser power was carefully limited to 0.1 mW during all measurements.

To elucidate the reaction mechanism and identify the oxygen source involved in ethylene glycol electrooxidation (EGOX), isotopic labeling experiments were carried out using H_2O^{18} enriched water. The electrolyte was prepared by replacing 10% of H_2O^{16} with H_2O^{18} to obtain an alkaline electrolyte (1 M KOH) containing 1 M ethylene glycol. HEOS deposited on carbon cloth was employed as the working electrode.

Chronoamperometry was conducted at a constant applied potential, and electrolyte samples were collected after 4, 6, and 10 h of electrolysis. Prior to ^{1}H NMR analysis, a required amount of $CHCl_3$ was added to the collected electrolyte samples to facilitate phase separation and suppress residual proton exchange effects. The liquid products were subsequently analyzed by ^{1}H NMR spectroscopy to identify and quantify reaction products and to assess the impact of isotopic labeling on product selectivity.

Ex situ Fourier-transform infrared (FTIR) spectra were recorded using a JASCO FT/IR-4600 spectrometer to investigate structural and vibrational changes in the catalyst. After

electrochemical testing, the HEOS catalyst was recovered by sonicating the carbon cloth electrode in an appropriate solvent to detach the deposited catalyst layer. IR spectra were recorded for the pristine HEOS, HEOS after electrochemical testing in the conventional H_2O^{16}-based electrolyte, and HEOS after the isotopic labeling experiment. Comparative analysis of these spectra was used to evaluate catalyst stability and to provide spectroscopic evidence supporting oxygen involvement in the proposed reaction pathway.

Electrochemical Measurements: Catalyst powder (5 mg) and 5 wt% Nafion solution (24 µL) were added to isopropyl alcohol (976 µL), and the mixture was ultrasonicated for 30 min in an ice–water bath to obtain a homogeneous catalyst ink. An appropriate amount of this ink was drop-cast onto carbon paper (Fuel Cell Earth, Japan) using a loading area of 0.7 cm × 0.7 cm (overall surface area = 0.49 cm^2) to afford a catalyst loading of ~0.5 mg cm^{-2}. The thus obtained working electrode was air-dried and mounted onto an electrode holder for electrochemical measurements.

Electrochemical tests were carried out in a conventional three-electrode cell using 1 M KOH or 1 M KOH + 1 M EG as the electrolyte. The working, reference, and counter electrodes corresponded to the catalyst-coated carbon paper, saturated Ag/AgCl electrode, and Pt wire (230 mm length × 0.5 mm diameter), respectively. All experiments were performed using an IviumStat electrochemical workstation (IVIUM Technologies B.V., the Netherlands). Cyclic voltammetry (CV) and linear sweep voltammetry measurements were conducted at a scan rate of 5 mV s^{-1} with an applied *iR* compensation of 90%. All measured potentials were converted to the reversible hydrogen electrode (RHE) scale using Equation (1):[1,2]

$$E(\mathrm{RHE})\ [\mathrm{V}] = E(\mathrm{Ag/AgCl})\ [\mathrm{V}] + 0.059\mathrm{pH} + 0.197. \quad (1)$$

The Tafel slope was determined from the steady-state region using Equation (2):[1,2]

$$\eta = a + b\log j, \quad (2)$$

where η is the overpotential (V), b is the Tafel slope (mV dec^{-1}), and j is the current density (mA cm^{-2}).

The electrochemically active surface area was estimated from the double-layer capacitance (C_{dl}, obtained by performing CV measurements within a nonfaradaic potential window). Electrochemical impedance spectroscopy (EIS) measurements were carried out at different applied potentials using sinusoidal voltage perturbations in the frequency range of 100 kHz to 1 mHz. The diameters of the semicircles in the resulting Nyquist plots were used to determine charge transfer resistance (R_{ct}). C_{dl} was calculated as the slope of the plot of the half difference in current density at the midpoint potential vs. the scan rate. Distribution of relaxation times (DRT) analysis provided further insights into the electrode kinetics, equivalent circuit behavior, and reaction mechanisms. DRT plots were generated by fitting EIS peaks using a Gaussian basis function with a regularization parameter of 0.1.[3] DRT analysis revealed frequency-resolved enhancements in EG electrooxidation over the high-entropy oxysulfide (HEOS) compared with that over the high-entropy oxide (HEO), reflecting the superior catalytic kinetics and mechanistic diversity of the former catalyst. The sharper intermediate-frequency peak (10–100 Hz) observed for HEOS signified accelerated C–C bond cleavage driven by S-stabilized O vacancies and high-valent metal centers (Cu^{2+}, Fe^{3+}, Co^{3+}), as supported by X-ray photoelectron spectroscopy (XPS) and electron paramagnetic resonance spectroscopy data. Enhanced OH turnover, indicated by a narrower high-frequency peak (>1 kHz), confirmed elevated AOM activity, which agreed with the increased number of surface states near the Fermi level revealed by valence-band XPS. At low frequencies (<1 Hz), HEOS exhibited broad peaks not observed for HEO, which suggested active lattice O mediation enabled by vacancy stabilization. Finally, the ultrahigh-frequency peak (>10 kHz) reflected the accelerated hydrogen atom transfer kinetics of HEOS, facilitated by improved surface covalency and metallicity due to S incorporation. Collectively, these features underscore the multifaceted advantages of HEOS in driving efficient EG oxidation through synergistic surface mechanisms. Chronoamperometric measurements were conducted at selected potentials to evaluate the long-term stability of the electrocatalyst.

Faradaic efficiency (FE) was calculated using Equation (3):[2]

FE (%) = 100% × (mol of product × n × F)/Q, (3)

where n = 6 is the number of electrons transferred during the glycol-to-formate conversion, Q is the total charge passed (C), and F is the Faraday constant (96,485 C mol^{-1}).

Product yield was calculated using Equation (4):[3]

Yield = (Q × FE)/(F × t × A × n), (4)

where t is the time elapsed before product sampling, and A is the geometric surface area of the working electrode. The FE for the glycol-to-formate conversion was evaluated in three- and two-electrode configurations. Q was determined by performing CV measurements in the three-electrode setup and chronoamperometric measurements in the two-electrode setup.

Calculation of FE from ^{1}H NMR Data: The stock (product) solution (1 mL) was spiked with chloroform (3.95 μL) as an internal standard. The concentration of chloroform (molecular weight = 119.378 g mol^{-1}, density = 1.49 g mL^{-1}) in the spiked solution was calculated as follows. $CHCl_3$ amount = 3.95 × 10^{-3} mL × 1.49 g mL^{-1}/119.378 g mol^{-1} = 4.93 × 10^{-5} mol. $CHCl_3$ concentration = 4.93 × 10^{-5} mol/10^{-3} L = 0.0493 M = 49.3 mM.

The product amount was calculated by integrating the ^{1}H NMR peaks and normalizing them against the peak intensity of the internal standard.

To identify oxidation products, we recorded the ^{1}H and ^{13}C NMR spectra of pure formic acid and formaldehyde in a consistent alkaline matrix (1 M KOH). Reference samples were prepared by dissolving formic acid or formaldehyde in 1 M KOH to a concentration of 0.1 M while maintaining the pH used in the electrooxidation experiments. The ^{1}H and ^{13}C shifts of the product peaks precisely matched those of the formic acid reference, which confirmed the formation of formate as the sole oxidation product. The absence of peaks corresponding to formaldehyde or other byproducts indicated the high selectivity of the EG oxidation process.

Thermodynamic Parameters of HEOS and HEO: The configurational entropy (ΔS_{con}), mixing enthalpy (ΔH_{mix}), atomic size mismatch (δ), average electronegativity ($\overline{\chi}$), electronegativity mismatch ($\Delta\chi$), and average valence electron concentration (VEC_{avg}) were calculated using Equation (5–11).[4–6]

$$\bar{r} = \sum x_i \,.r_i, \tag{5}$$

where $\bar{r}$ (pm) is the average atomic radius, r_i (pm) is the radius of element i, and x_i is the atomic percentage of element i.

$$\delta\ (\%) = \sqrt[2]{\sum x_i \left(1 - \frac{r_i}{\bar{r}}\right)^2}, \tag{6}$$

The high value of δ suggests a large lattice distortion typical of HEOs rather than metallic solid solutions.

$$\overline{\chi} = \sum x_i \chi_i, \tag{7}$$

where χ_i is the electronegativity of element i.

$$\Delta\chi = \sqrt{\sum x_i (\chi_i - \overline{\chi})^2}, \tag{8}$$

A high $\Delta\chi$ typically favors compound formation over a metallic solid solution.

$$\mathrm{VEC}avg = \sum x_i \,\mathrm{VEC}_i, \tag{9}$$

where VEC_i is the VEC of element i. This value is borderline between BCC and FCC preferences for metals; however, in oxides, it reflects electron density rather than phase stability directly.

$$\Delta H_{\mathrm{mix}} = \sum 4\Delta H_{ij}^{\mathrm{mix}}\ x_i x_j, \tag{10}$$

where x_i and x_j are the atomic percentages of elements i and j, respectively, and $\Delta H^{mix}{}_{ij}$ is the mixing enthalpy of their binary alloy pair.

$$\Delta Scon = -R \sum x_i \ln x_i, \tag{11}$$

where R is the universal gas constant (8.314 J mol^{-1} K^{-1}). Configurational entropy is a key thermodynamic parameter contributing to HEO and HEOS stabilization by minimizing free energy and favoring uniform elemental distributions.

Table S1. Elemental composition of the high-entropy oxide (HEO) determined by scanning transmission electron microscopy coupled with energy-dispersive X-ray spectroscopy (STEM-EDX).[7]

**Element**	**Content (at%)**	**Atomic fraction (*x*)**
O	36.9	0.369
Mn	11.20	0.1120
Fe	14.43	0.1443
Co	14.12	0.1412
Ni	11.48	0.1148
Cu	11.88	0.1188

Table S2. Elemental composition of the high-entropy oxysulfide (HEOS) determined by STEM-EDX.

**Element**	**Content (at%)**	**Atomic fraction (*x*)**
O	48.3	0.483
S	4.08	0.0408
Mn	9.30	0.0930
Fe	9.09	0.0909
Co	9.71	0.0971
Ni	9.75	0.0975
Cu	9.77	0.0977

Table S3. Melting points of the transition metals contained in HEO and HEOS.[8]

**Metal**	**Melting point (K)**
Mn	1519
Fe	1811
Co	1768
Ni	1728
Cu	1358

O and S were excluded from melting point and mixing enthalpy calculations, as they are nonmetals and do not form alloy phases.

Table S4. Binary mixing enthalpies of transition metal alloys.[9]

**Binary pair**	**ΔH_{mix} (kJ mol^{-1})**
Mn–Fe	0
Mn–Co	−5
Mn–Ni	−8
Mn–Cu	4
Fe–Co	−1
Fe–Ni	−2
Fe–Cu	13
Co–Ni	0
Co–Cu	4
Ni–Cu	4

Table S5. Thermodynamic parameters calculated for HEO and HEOS.

**Parameter**	**ΔS_{con} (J mol^{-1} K^{-1})**	**δ (%)**	**VEC_{avg}**	**$\Delta\chi$**	**$\bar{r}$ (pm)**	**ΔH_{mix} (kJ mol^{-1})**	**Ω**
HEO	13.89	31.3	7.88	0.789	101.6	1.623	1.29
HEOS	13.32	34.77	7.45	0.799	93.08	1.475	1.18

Table S6. Performances of different catalysts for ethylene glycol (EG) oxidation in alkaline media.

Catalyst	Electrolyte	Stability	Main product and FE	Ref.
FeCoNi/C	1.0 M KOH + 1.0 M EG	95%@0.14 h	Glycolate (~36%), oxalate (~43.5%)	10
$NiSe_2$/C	1.0 M KOH + 1 M EG	45.4%@10 h	Formate (83.4%), oxalate (5.4%), glycolate (7.4%)	11
rGO-NiMn	1.0 M KOH + 1 M EG	26.3%@2 h	Oxalate (n.a.)	12
NiCu60s/NF	1 M KOH + 0.3 M EG	~33%@1 h	Formate (n.a.), glycolate (n.a.)	13
Co-Ni/CP	1 M KOH + 1 M EG	n.a@24 h	Glycolate (96.4%)	14
Ni-Co-S NSAs/NF	1 M KOH + 1 M EG	~40%@24 h	Formate (90%)	2
Ni/Cu/C	0.1 M KOH + 0.1 M EG	n.a.	n.a.	15
(PtIr)(FeMoBi)	1 M KOH + 1 M EG	n.a	Glycolate (94–100%)	1
PdAg/NF	0.5 M KOH + 1 M EG	n.a	Glycolate (83.4%)	16
Pd/MWCNT-COOH	2 M KOH + 5 wt% EG	n.a	Carbonate (26%), formate (4%), oxalate (5%), and glycolate (65%)	17
HEOS	1 M KOH + 1 M EG	94%@20 h	Formate (84.6%)	This work

Table S7. Double layer capacitances (C_{dl}) and electrochemically active surface areas (ECSAs) of HEOS and HEO.

Catalyst	C_{dl} (mF cm^{-2})	ECSA (cm^2)
HEO	5.38	134.5
HEOS	12.73	318.25

Table S8. Cell voltages of various electrochemical coupling systems.

**Electrocatalyst**	**Reaction**	**Electrolyte**	**Cell voltage**	**Ref.**
Pt/Rh metallene‖Pt/Rh metallene	EGOR‖HER	1 M KOH + 3 M EG	0.51 V@10 mA cm^{-2}	18
$PtRh_{0.02}$@Rh NWs‖$PtRh_{0.02}$@Rh NWs	EGOR‖HER	EGOR: 0.1 M KOH + 1 M EG<br>HER: 0.1 M KOH	0.66 V@10 mA cm^{-2}	19
CuO/Co_3O_4‖CuO/Co_3O_4	EGOR‖NO_2RR	EGOR: 1 M KOH + 0.1 M EG<br>NO_2RR: 1 M KOH + 0.1 M $NaNO_2$	1.52 V@10 mA cm^{-2}	20
Cu(F)@CuO@$Ni(OH)_2$‖Cu(F)@Pb-SnO	EGOR‖CO_2RR	EGOR: 1 M KOH + 0.1 M EG<br>CO_2RR: 0.5 M $KHCO_3$	1.63 V@10 mA cm^{-2}	21
Co-Ni_3N/CC‖Co-Ni_3N/CC	EGOR‖HER	PET hydrolysate (7.68 ppm terephthalate + 3.48 EG)	1.46 V@50 mA cm^{-2}	22
$NiSe_2$‖Pt	EGOR‖HER	1 M KOH + 1 M EG	1.395 V@50 mA cm^{-2}	11
CoP–Cu_3P/CC‖CoP–Cu_3P/CC	EGOR‖HER	1 M KOH + 0.1 M EG	1.21 V@10 mA cm^{-2}	23
PdAg/NF‖Pt plate	EGOR‖HER	0.5 M KOH + 1 M EG	1.02 V@20 mA cm^{-2}	16
Pt/Ir‖Pt/Ir	EGOR‖HER	EGOR: 1 M KOH + 1 M EG<br>HER: 1 M KOH	0.36 V@10 mA cm^{-2}<br>0.61 V@100 mA cm^{-2}	24
HEOS‖Pt/C	EGOR‖HER	EGOR: 1 M KOH + 1 M EG<br>HER: 1 M KOH	1.37 V@10 mA cm^{-2}	This work

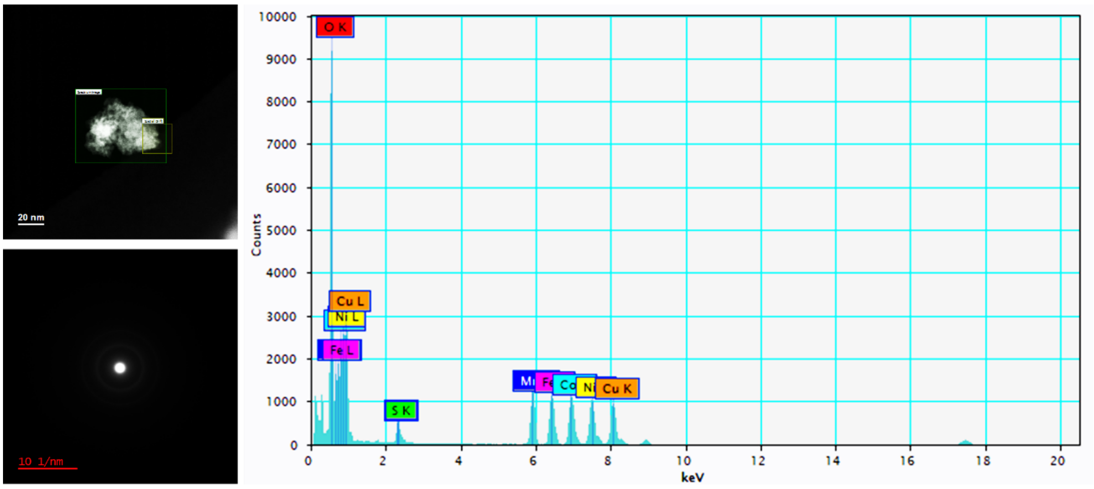

Figure S1. Selected area electron diffraction (SAED) pattern and energy-dispersive X-ray spectroscopy (EDS) profile of HEOS. The SAED pattern displays diffraction rings indicative of polycrystalline domains and successful phase formation. The EDS profile reveals the uniform distribution of constituent transition metals and S, supporting compositional homogeneity and successful S incorporation into the HEO framework.

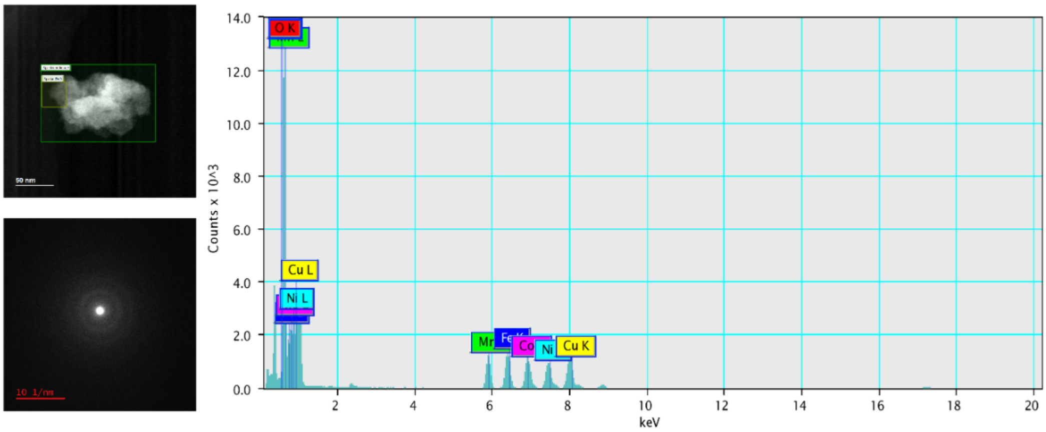

Figure S2. SAED pattern and EDS profile of HEO. The SAED pattern reveals a polycrystalline nature and features well-defined diffraction rings indicative of a multiphase cubic structure. The EDS profile reveals the homogeneous distribution of constituent transition metals, indicating compositional uniformity and successful element integration into the HEO matrix.

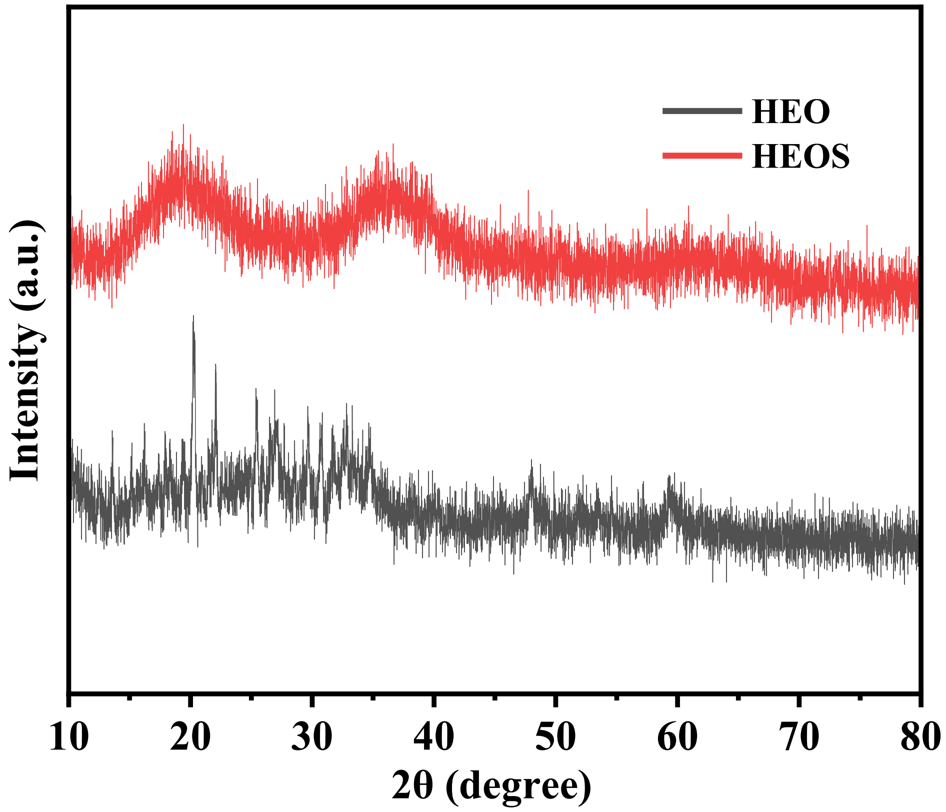

Figure S3. X-ray diffraction patterns of HEOS and HEO confirming phase formation and crystallinity. The diffraction peaks correspond to a multiphase cubic structure, with comparable lattice parameters observed for both materials. The absence of impurity phases indicates that S was incorporated into the HEO lattice without altering the overall phase symmetry. The HEOS sample exhibits significantly enhanced diffraction intensity with prominent broad peaks at approximately 20° and 35° 2θ, indicating structural modification upon sulfur incorporation. The diffraction patterns demonstrate distinct differences in crystallinity and phase composition between the two materials.

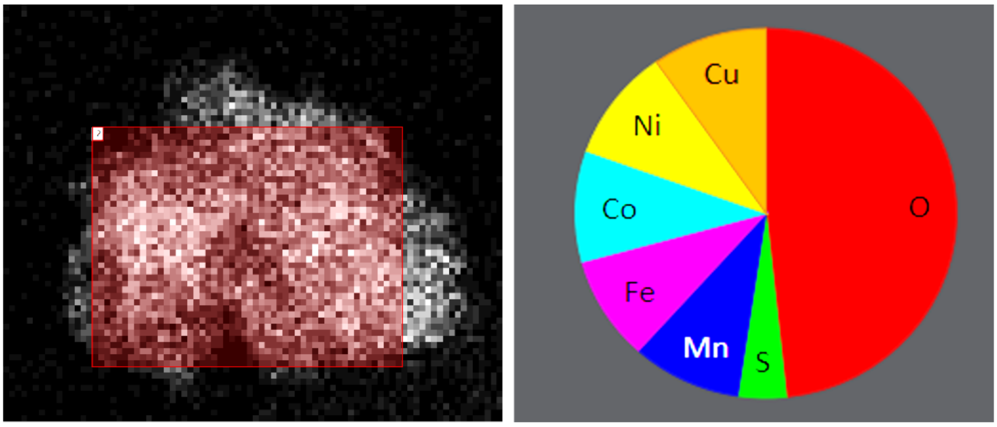

	Comp. (at%)	+/-
O	48.3	0.2
S	4.08	0.05
Mn	9.30	0.06
Fe	9.09	0.06
Co	9.71	0.06
Ni	9.75	0.06
Cu	9.77	0.06

Figure S4. Elemental composition of HEOS determined by STEM–EDX analysis. The data confirm the homogeneous incorporation of transition metals and S and support the configurational uniformity and successful synthesis of the high-entropy phase.

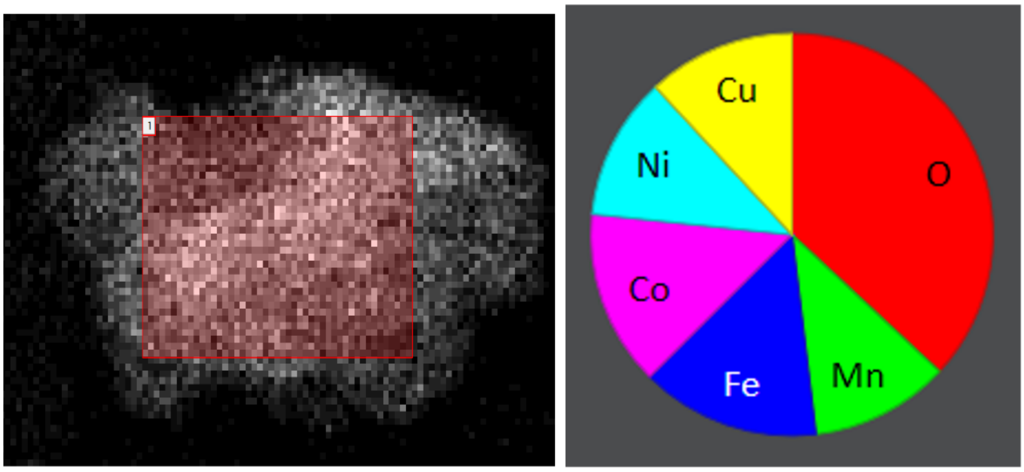

		Comp. (at%)	+/-
	O	36.9	0.4
	Mn	11.20	0.11
	Fe	14.43	0.13
	Co	14.12	0.12
	Ni	11.48	0.11
	Cu	11.88	0.11

Figure S5. Elemental composition of HEO determined by STEM–EDX analysis. The data confirm the uniform elemental distribution and successful incorporation of all target metal species and O into the high-entropy matrix, supporting the synthesis of a compositionally stable HEO framework.

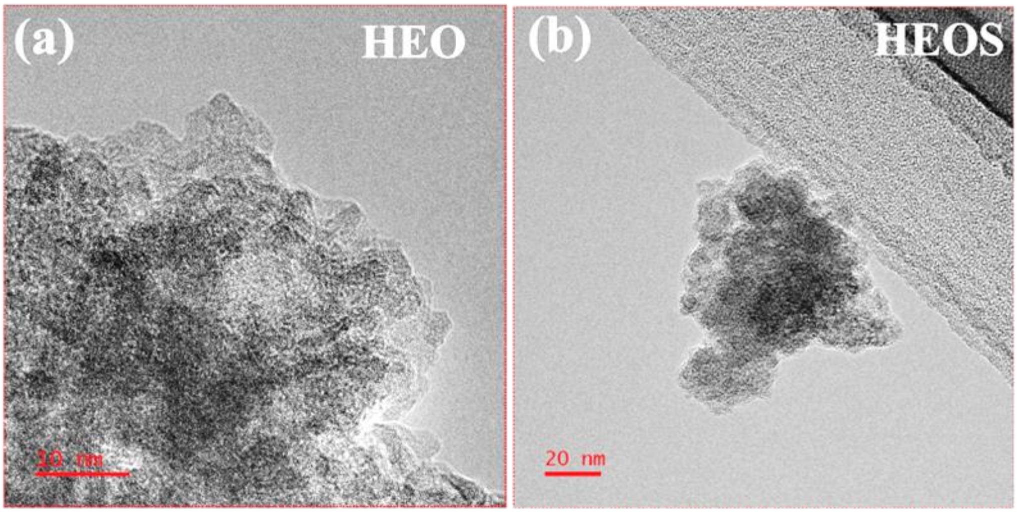

Figure S6. High-resolution transmission electron microscopy images of (a) HEO and (b) HEOS revealing lattice fringes indicative of high crystallinity in both cases. HEOS displays an enhanced defect density and minimally distorted lattice structures, consistent with S incorporation and O vacancy formation, whereas HEO exhibits a more uniform lattice alignment. These structural features support the important role of defect engineering in modulating catalytic activity.

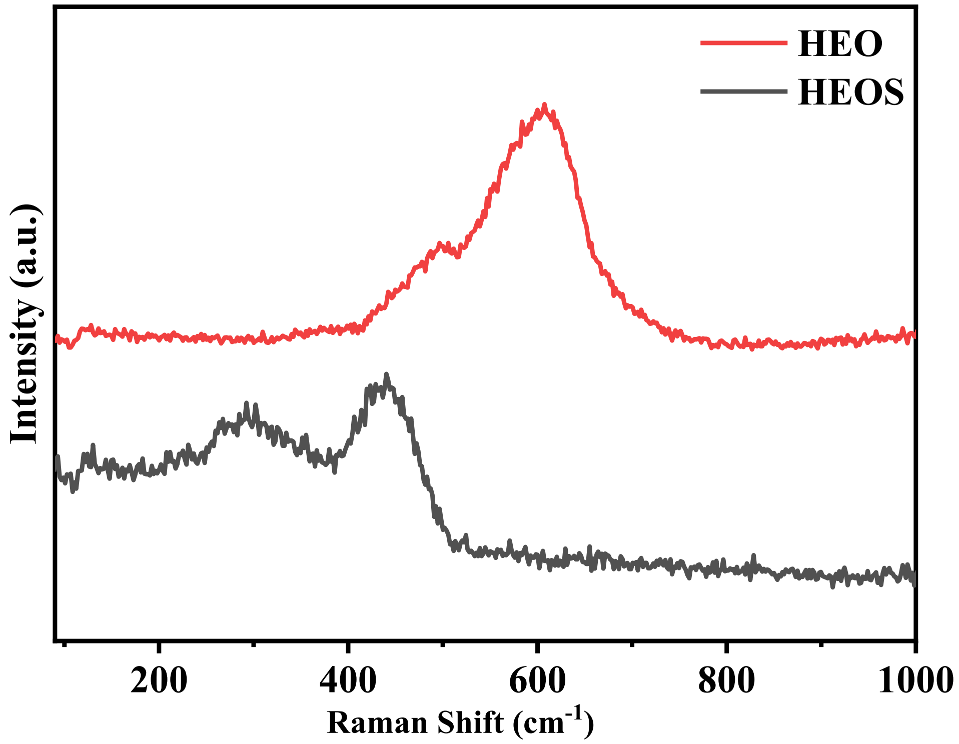

Figure S 7. Raman spectra of HEOS and HEO, representing the presence of M-S and M-O bond.

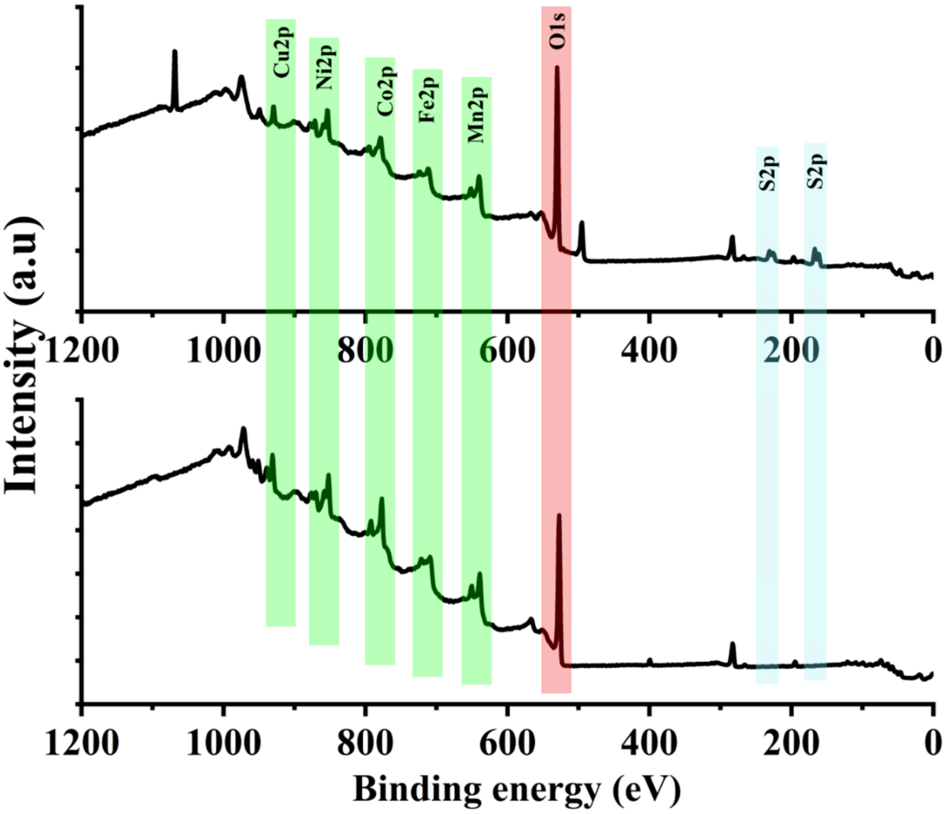

Figure S8. X-ray photoelectron survey spectra of HEO (bottom) and HEOS (top). The spectra confirm the presence of transition metals and O in HEO and additional S in HEOS, indicating successful S incorporation into the high-entropy matrix.

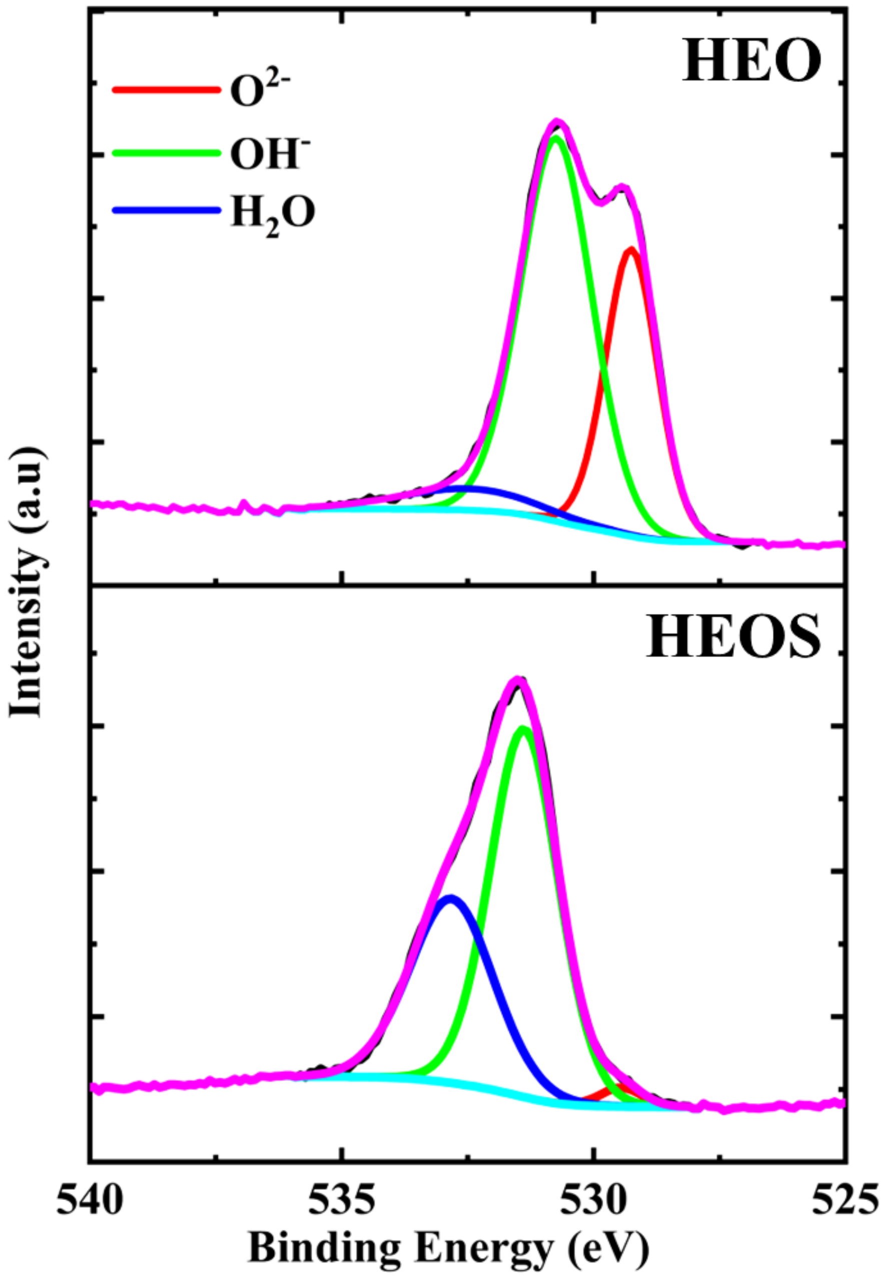

Figure S9. Deconvoluted O 1s X-ray photoelectron spectra of HEO and HEOS. For HEO, the dominant peaks correspond to lattice O (O^{2-}) and adsorbed hydroxyl groups. For HEOS, the relative intensity of O vacancy–related components increases because of S incorporation, suggesting enhanced lattice disorder and vacancy concentration. These spectral features corroborate the defect-mediated electronic structure modulation and support the synergistic role of S–O vacancies in catalytic activity enhancement.

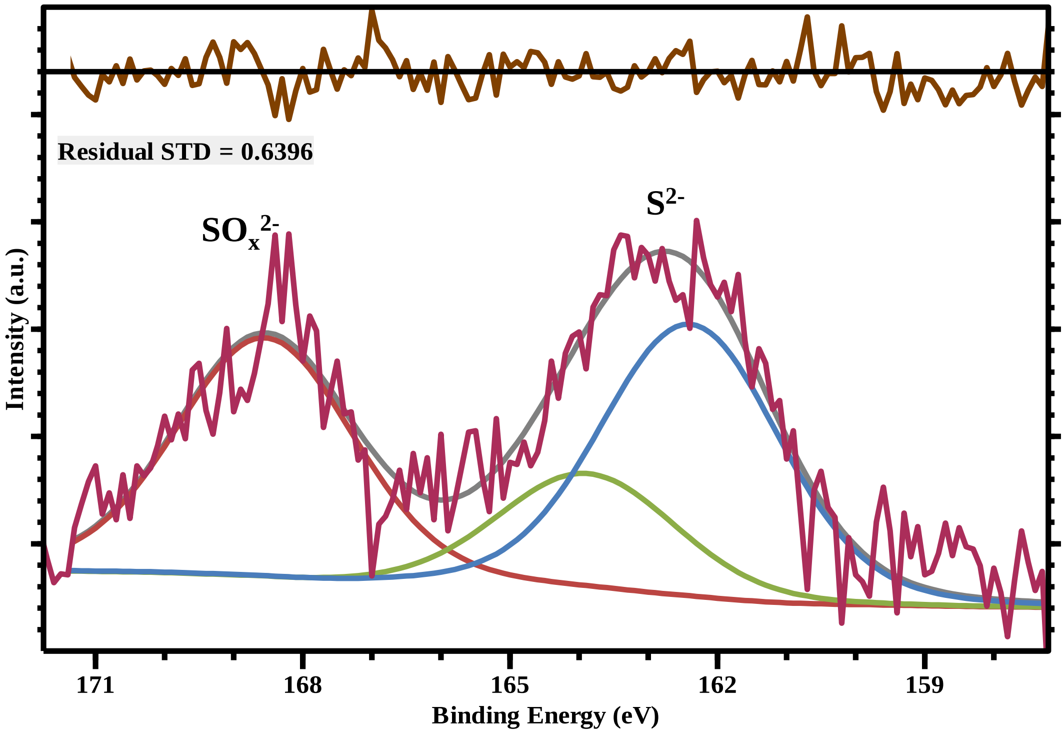

Figure S10. Deconvoluted S 2p X-ray photoelectron spectrum of HEOS (Residual STD 0.6396). The spectrum reveals S $2p_{3/2}$ and S $2p_{1/2}$ doublets, indicating the presence of both lattice-incorporated sulfide species (S^{2-}) and surface-bound S moieties. The dominant low-binding-energy component reflects strong covalent metal–S bonding, while higher-energy contributions suggest oxidized S species or partial sulfate formation. These features confirm S integration and support its role in electronic structure modulation and vacancy stabilization within the HEOS lattice.

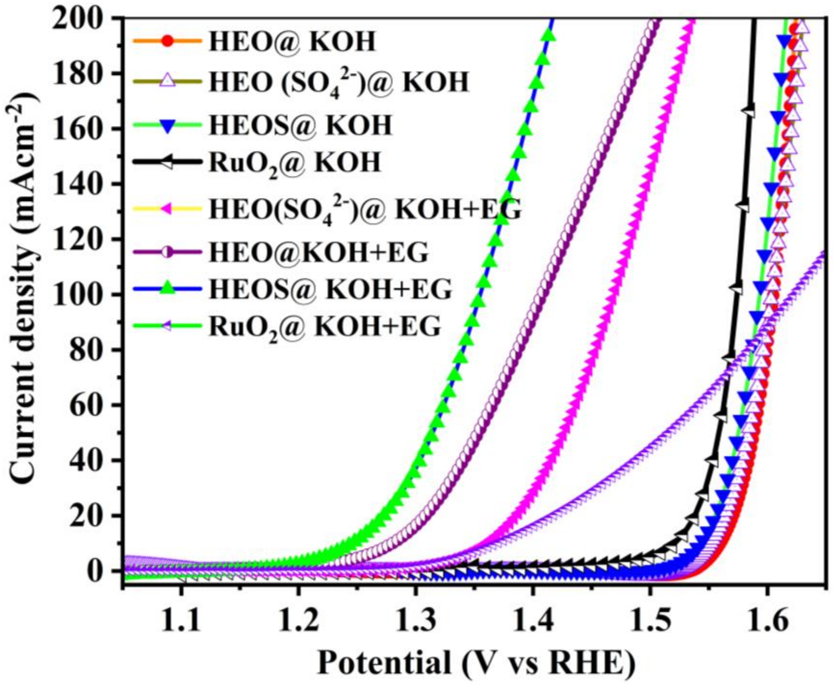

Figure S11. Linear sweep voltammograms of different EG oxidation electrocatalysts recorded in 1 M KOH. The curves reveal distinct onset potentials and current densities, with HEOS exhibiting a lower onset potential and higher catalytic current and thus showing superior electrocatalytic activity for EG oxidation. Sulfate-functionalized HEO (HEO(SO_4^{2-})) shows relatively sluggish kinetics.

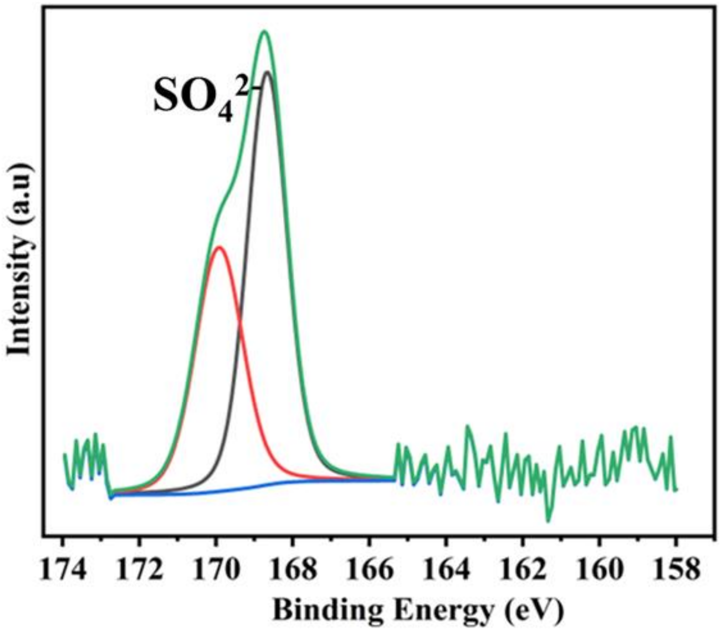

Figure S12. Deconvoluted S 2p X-ray photoelectron spectrum of HEO(SO_4^{2-}) showing characteristic S $2p_{3/2}$ and S $2p_{1/2}$ doublets at higher binding energies, consistent with surface-bound sulfate. These peaks distinguish oxidized S states from lattice-incorporated sulfide (S^{2-}) and confirm the presence of terminal sulfate groups. The absence of low-binding-energy components suggests the minimal incorporation of reduced S species, supporting the chemical nature of surface modification via sulfate functionalization.

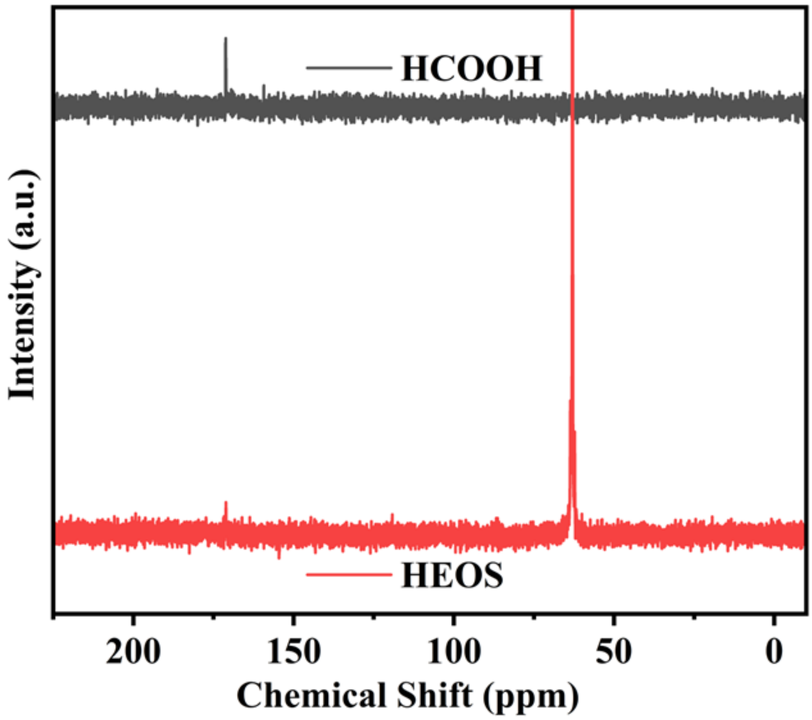

Figure S13. ^{13}C NMR spectra of the products of HEOS-catalyzed EG electrooxidation and reference formic acid in 1 M aqueous KOH. The former spectrum shows a resonance matching that of the formic acid reference. The absence of additional peaks confirms selective formate formation under alkaline conditions. The inclusion of the internal standard enables the quantitative comparison of product concentrations and spectral assignment validation.

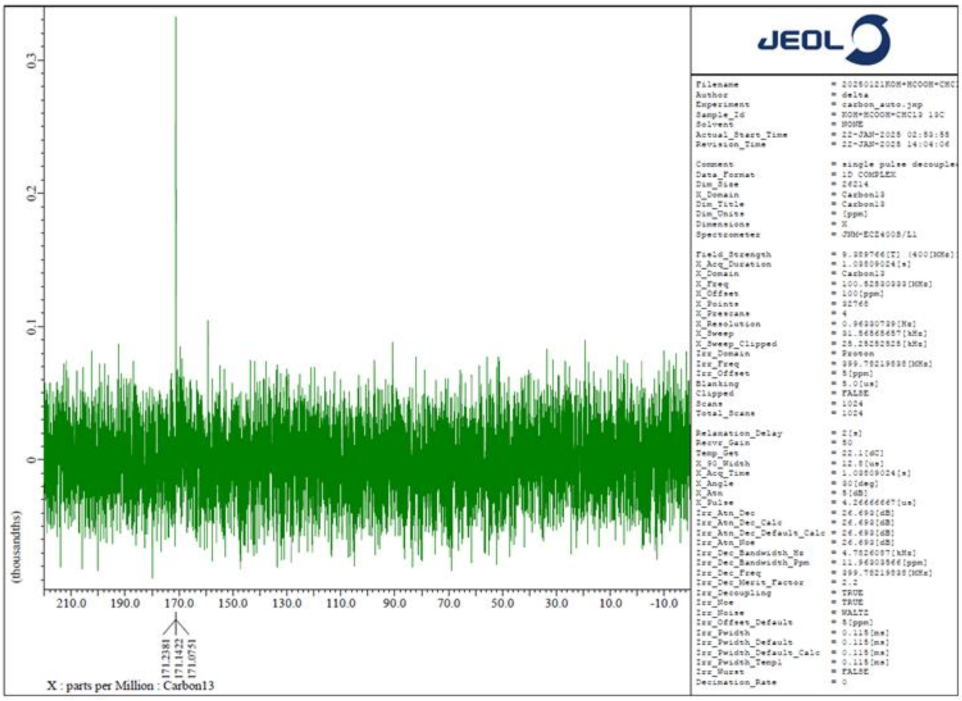

Figure S14. Integrated ^{13}C NMR spectrum of pure formic acid in 1 M aqueous KOH.

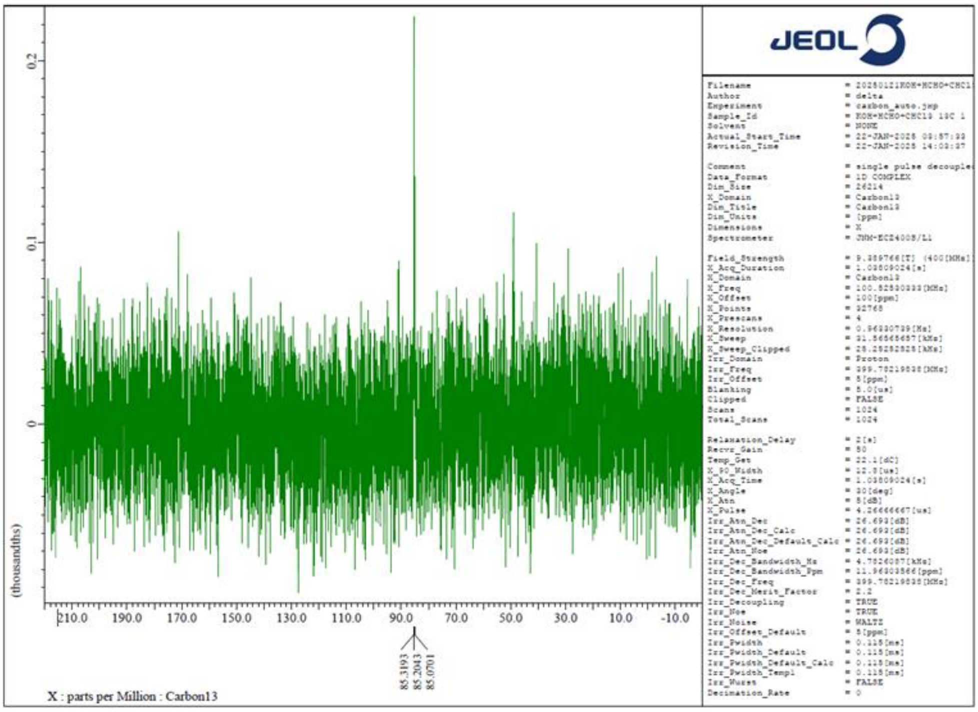

Figure S15. Integrated ^{13}C NMR spectrum of the product of HEOS-catalyzed EG electrooxidation. The spectrum displays a dominant signal corresponding to the carbonyl carbon of formate, confirming the selective conversion of EG to formic acid.

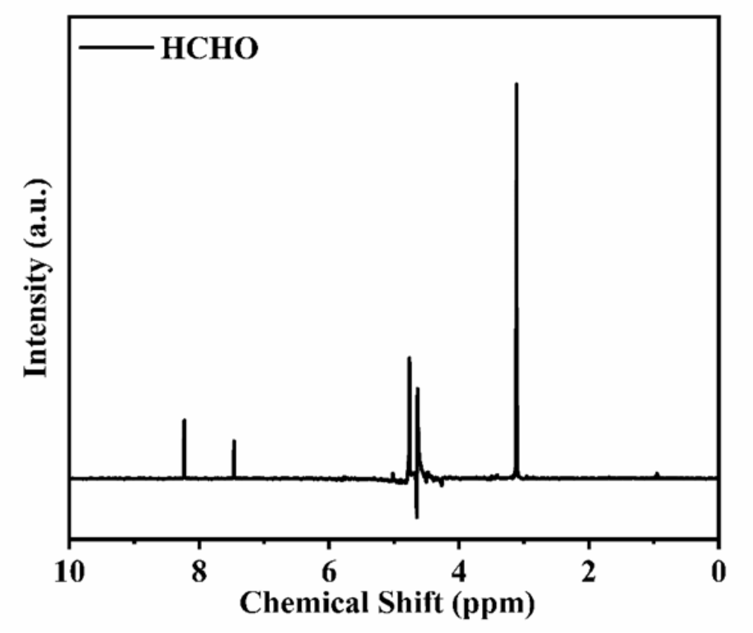

Figure S16. ^{1}H NMR spectrum of formaldehyde recorded in 1 M KOH + 1 M EG using $CHCl_3$ as an internal standard. The spectrum displays characteristic formaldehyde peaks, enabling comparative analysis across different alkaline media. Signal shifts and relative intensities reflect the chemical environment and reactivity of formaldehyde under alkaline conditions, with $CHCl_3$ providing a stable reference peak for quantitative calibration.

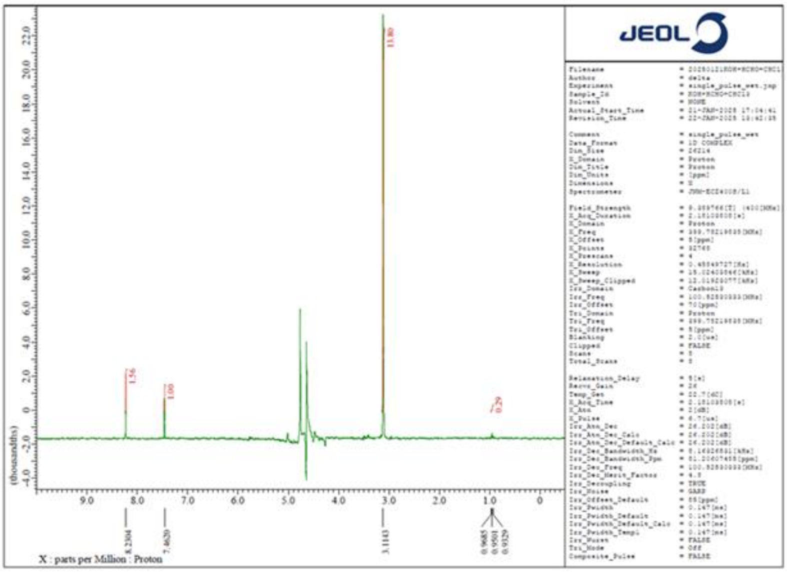

Figure S17. Integrated ^{1}H NMR spectrum of formaldehyde recorded in 1 M KOH using $CHCl_3$ as an internal standard.

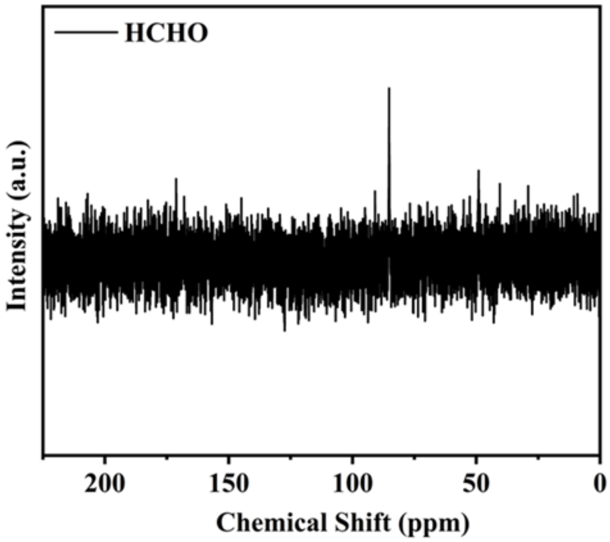

Figure S18. ^{13}C NMR spectrum of pure formaldehyde recorded in 1 M aqueous KOH.

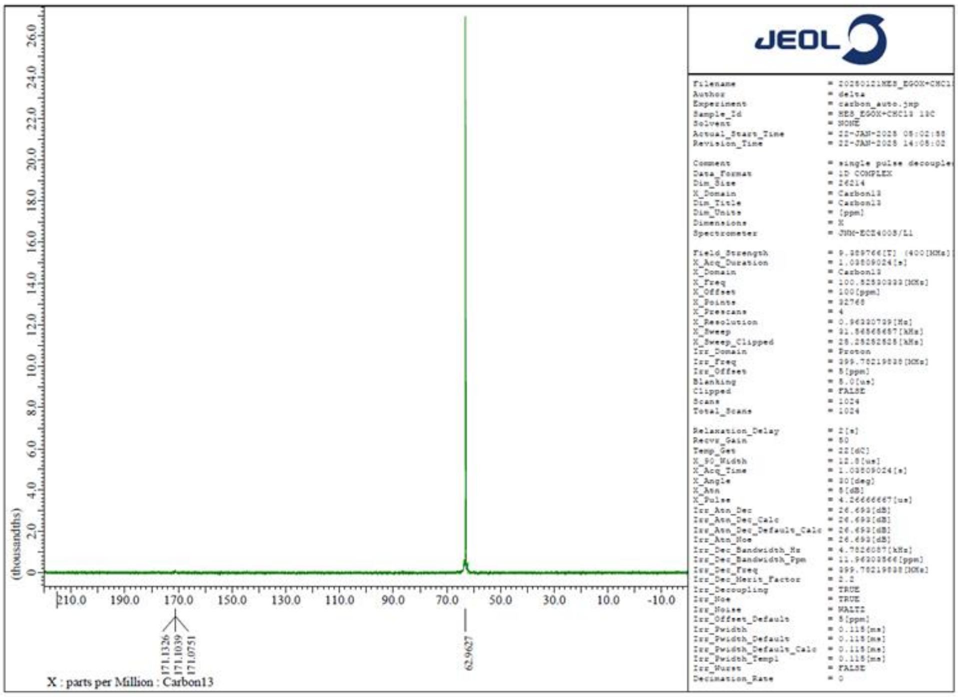

Figure S19. Integrated ^{13}C NMR spectrum of pure formaldehyde recorded in 1 M aqueous KOH.

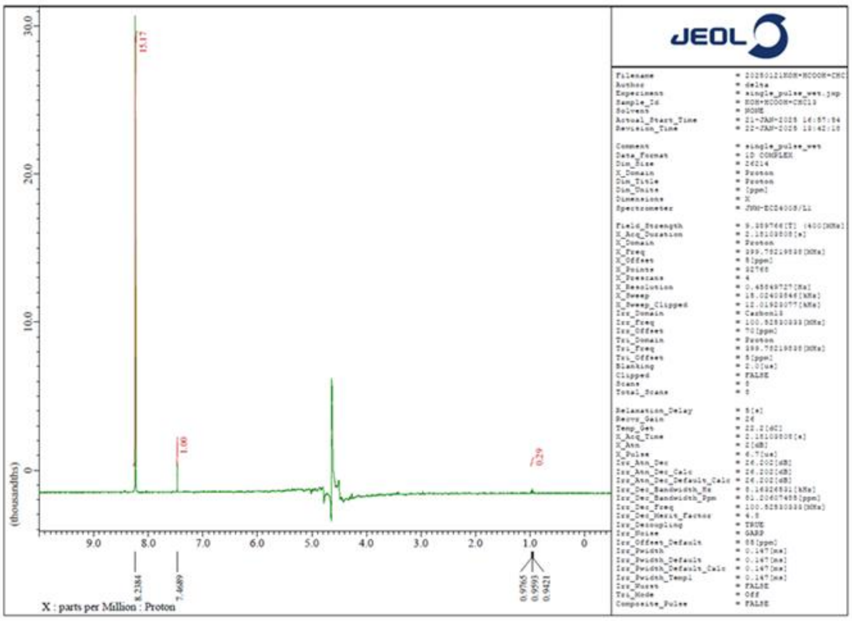

Figure S20. Integrated ^{1}H NMR spectrum of formic acid recorded in 1 M KOH using $CHCl_3$ as an internal standard.

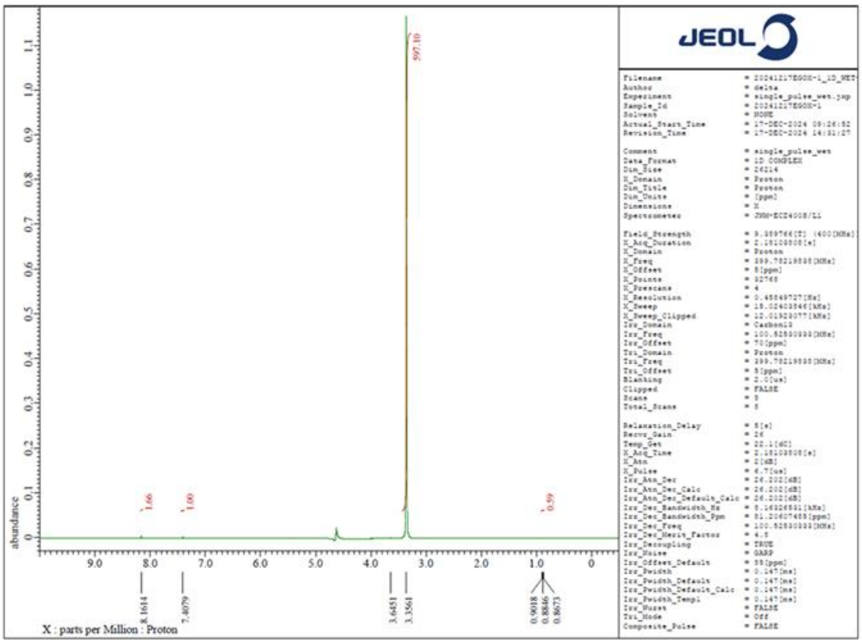

Figure S21. Integrated ^{1}H NMR spectrum of the product of HEOS-catalyzed EG oxidation in 1 M KOH + 1 M EG recorded using $CHCl_3$ as an internal standard. The spectrum features a singlet corresponding to the formyl proton of formic acid, confirming the selective conversion of EG to formate. The absence of additional peaks suggests minimal byproduct formation.

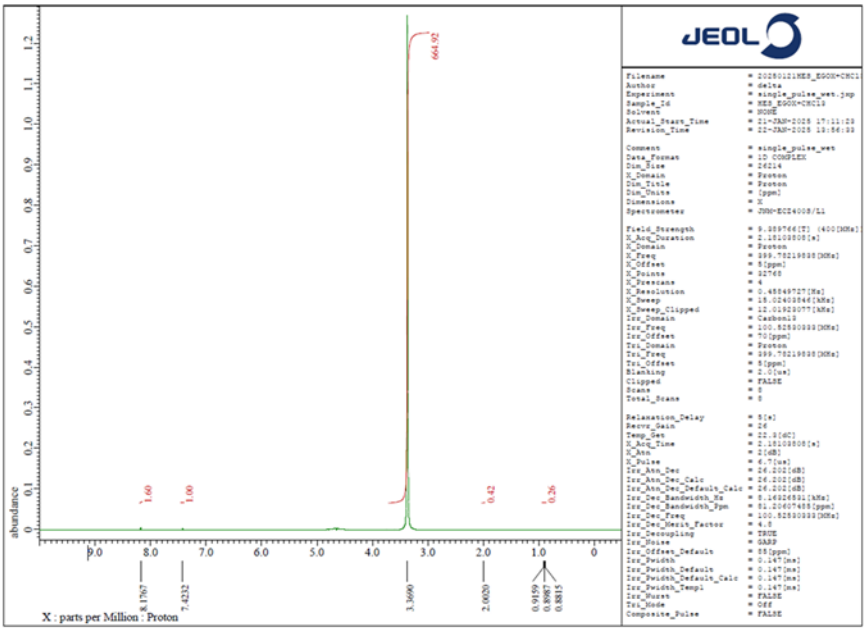

Figure S22. Integrated ^{1}H NMR spectrum of the product of HEO-catalyzed electrooxidation recorded in 1 M KOH + 1 M EG using $CHCl_3$ as an internal standard.

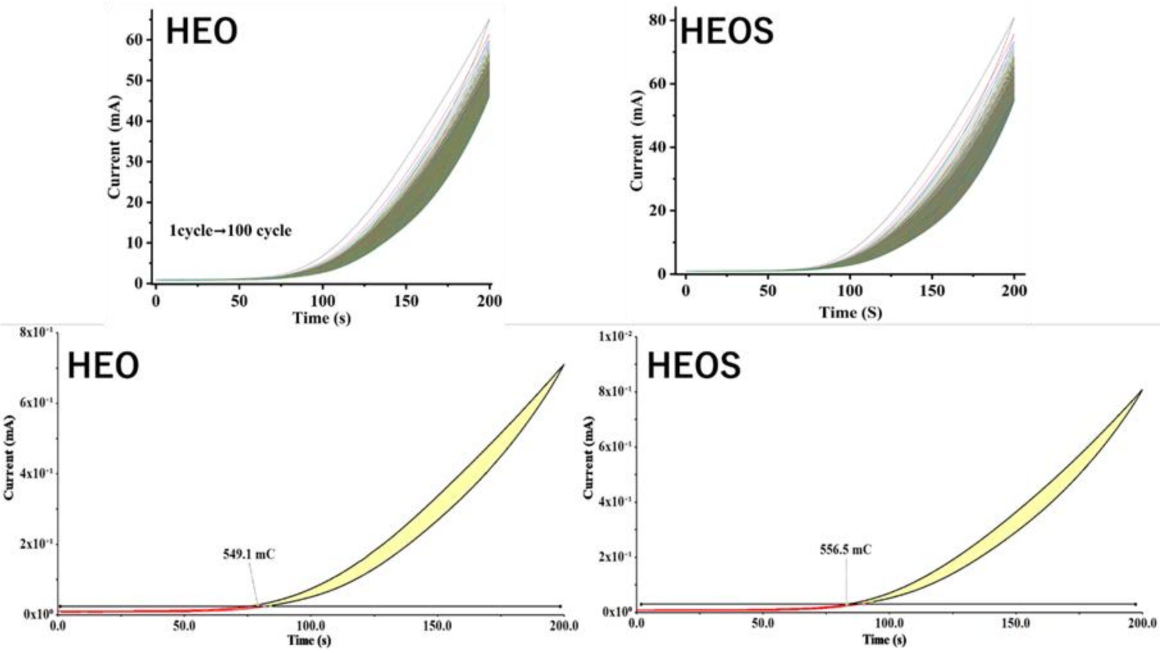

Figure S23. Cyclic voltammograms recorded over 100 consecutive cycles and the corresponding acquired charge (Q) per cycle for HEOS and HEO electrodes.

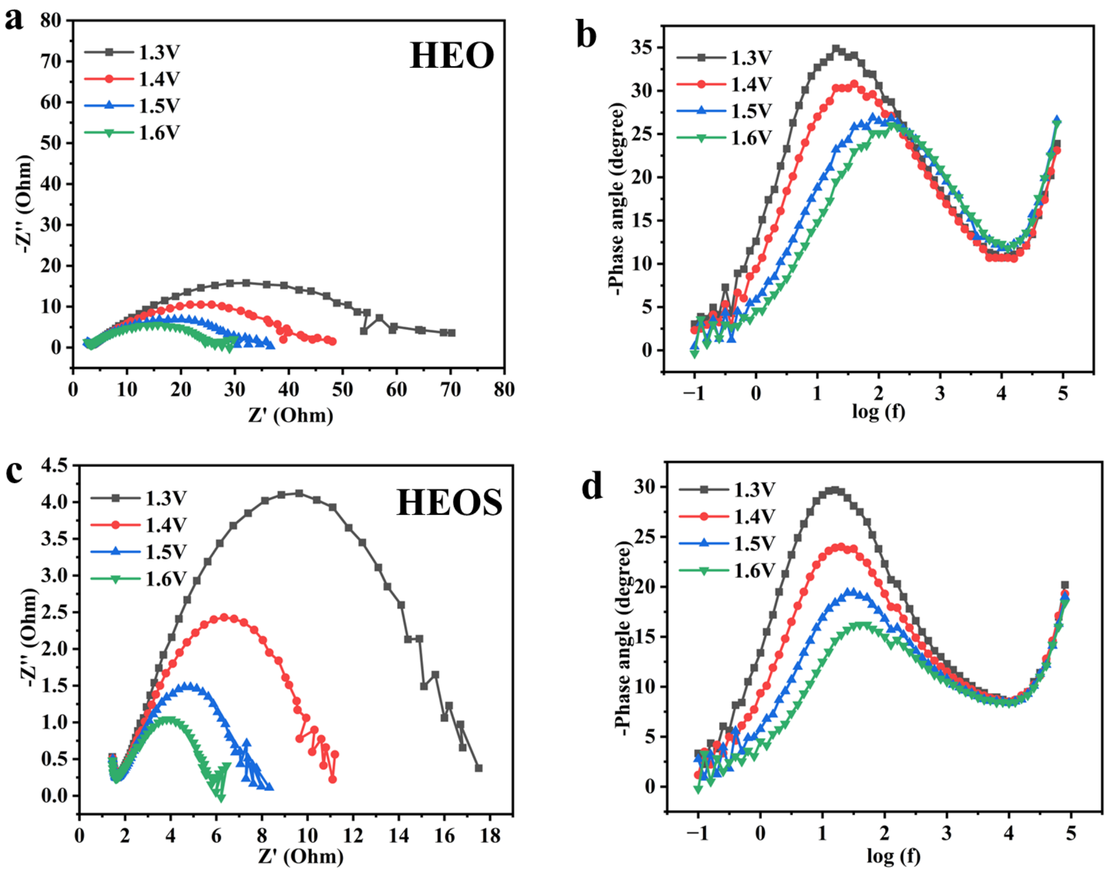

Figure S24. (a, c) Nyquist and (b, d) Bode phase angle plots for (a, b) HEO and (c, d) HEOS.

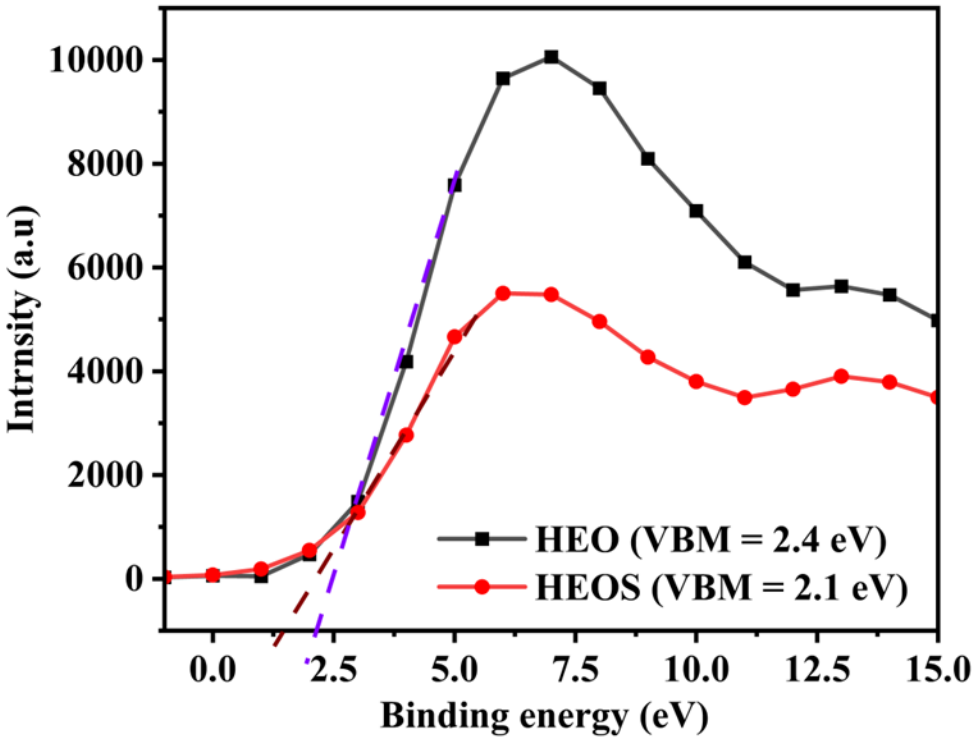

Figure S25. Valence band X-ray photoelectron spectra of HEO and HEOS. The spectra reveal differences in valence band edge positions, reflecting the electronic structure modulation induced by S incorporation. HEOS exhibits a shallower valence band maximum than HEO, which indicates an altered charge distribution and enhanced electronic conductivity. These shifts support the synergistic role of S and O vacancies in tailoring the density of states for improved electrocatalytic activity.

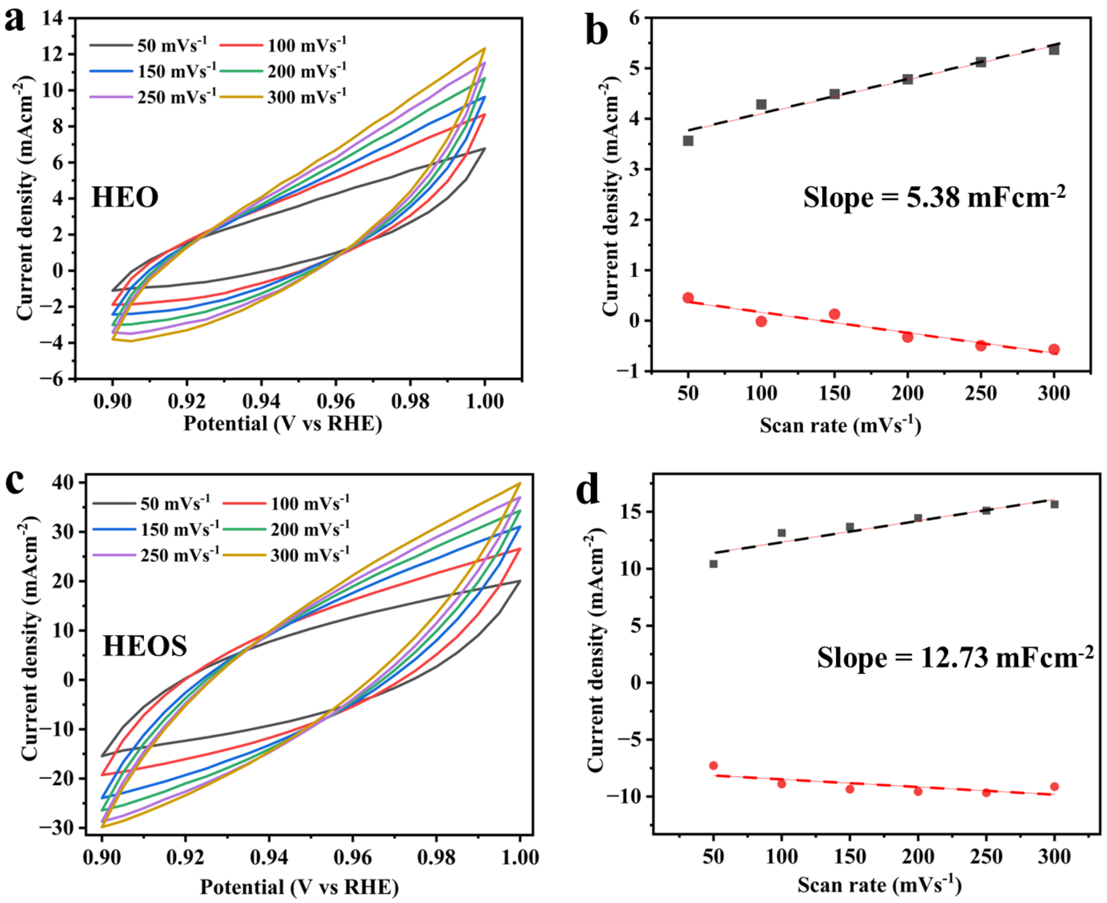

Figure S26. Cyclic voltammograms of (a) HEO and (c) HEOS recorded at varying scan rates and (b, d) corresponding linear fits of anodic/cathodic current–scan rate plots.

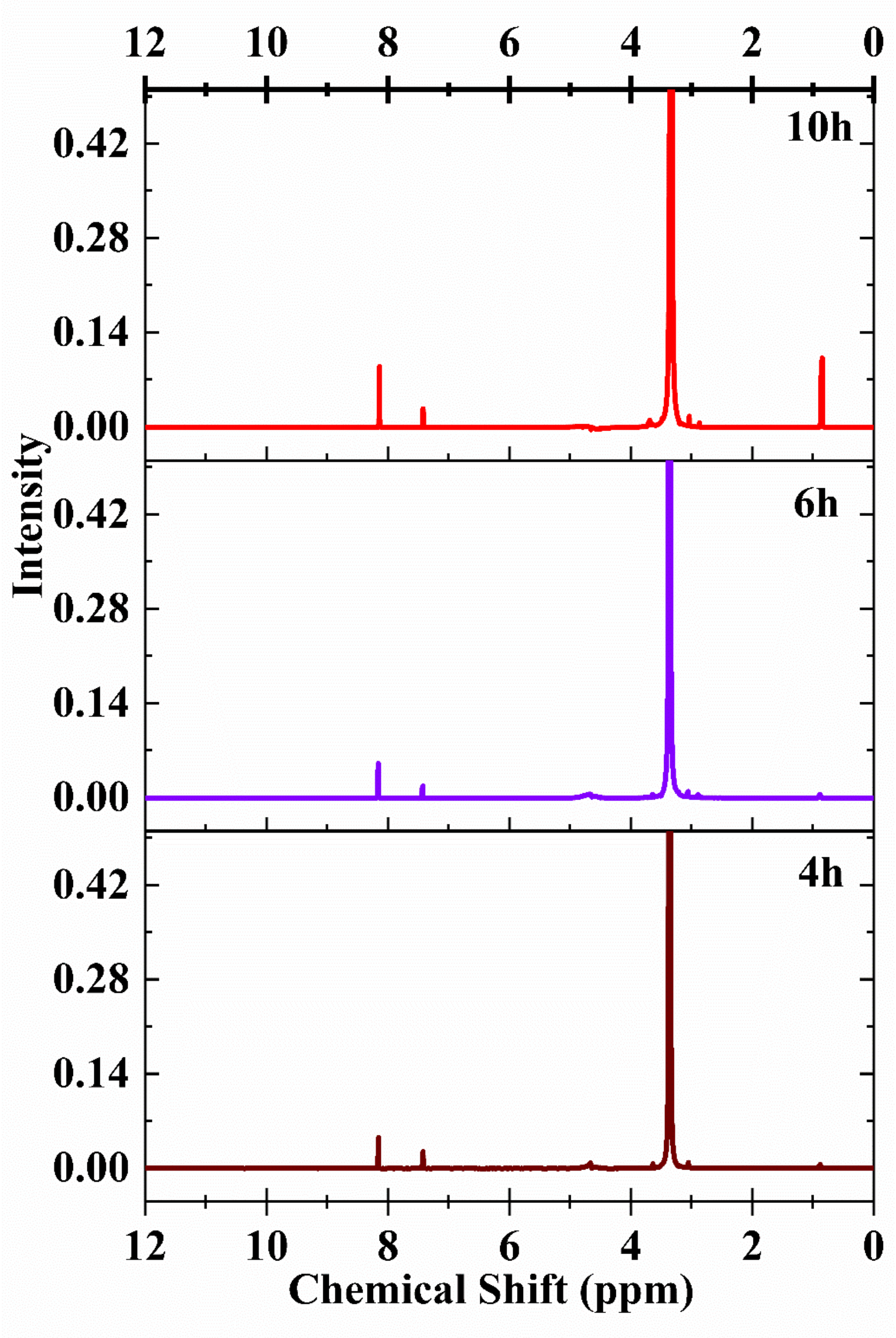

Figure S27. ^{1}H NMR spectra of the electrolyte collected during ethylene glycol electrooxidation over HEOS after 4, 6, and 10 h of chronoamperometric operation. The dominant resonance at ~8.15 ppm corresponds to formate, confirming its formation as the primary liquid product throughout the reaction, while the absence of additional proton signals indicates high product selectivity and stability over prolonged electrolysis.

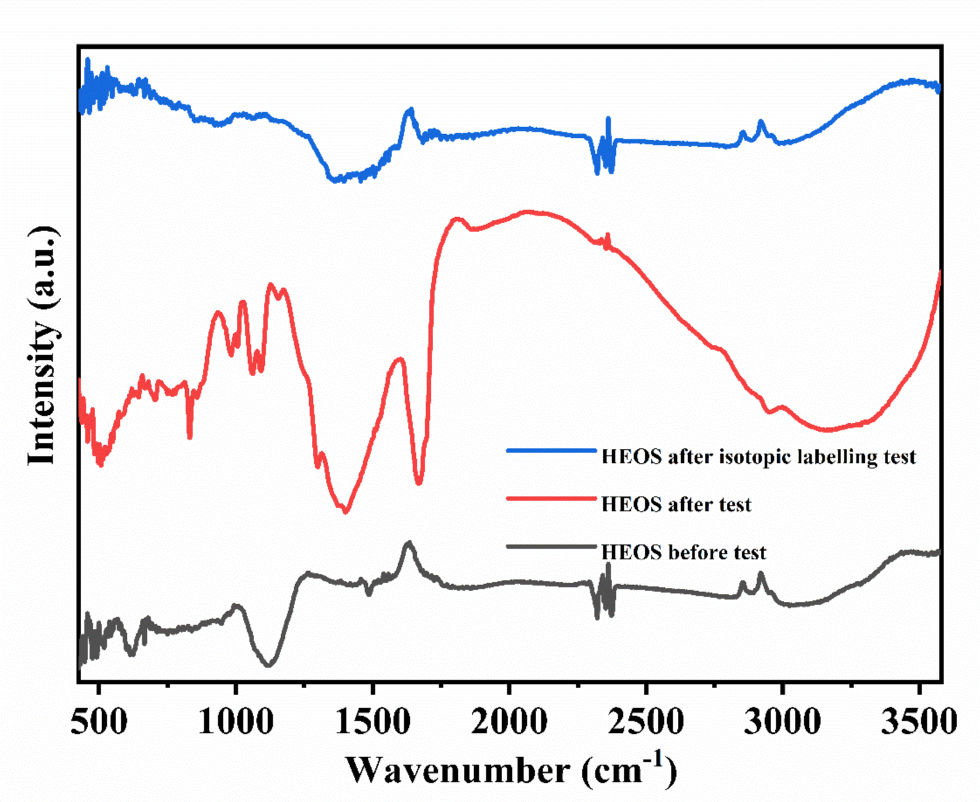

Figure S28. Fourier-transform infrared (FTIR) spectra of HEOS collected before electrochemical testing, after electrochemical testing, and after the isotopic labeling experiment. The comparison highlights the evolution of surface vibrational features upon electrochemical operation and confirms the structural integrity of HEOS following the $H_2{}^{18}O$-assisted isotopic labeling test.

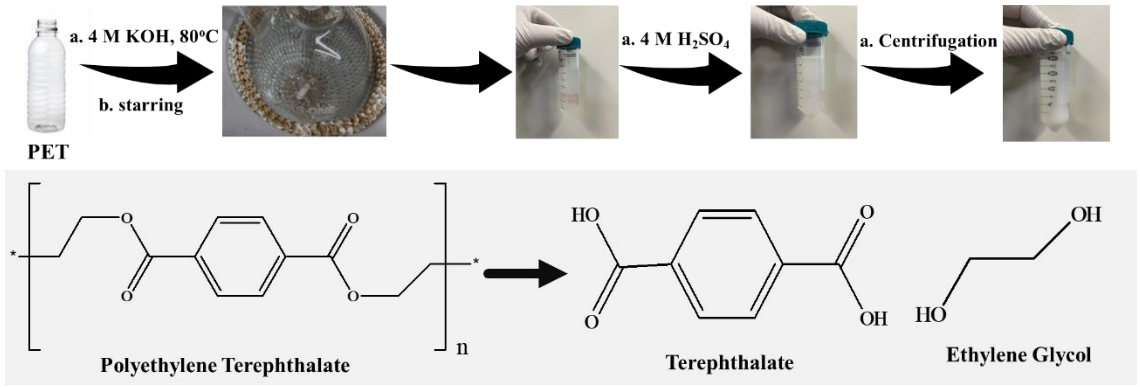

Figure S29. Schematic of polyethylene terephthalate (PET)-derived EG recycling.

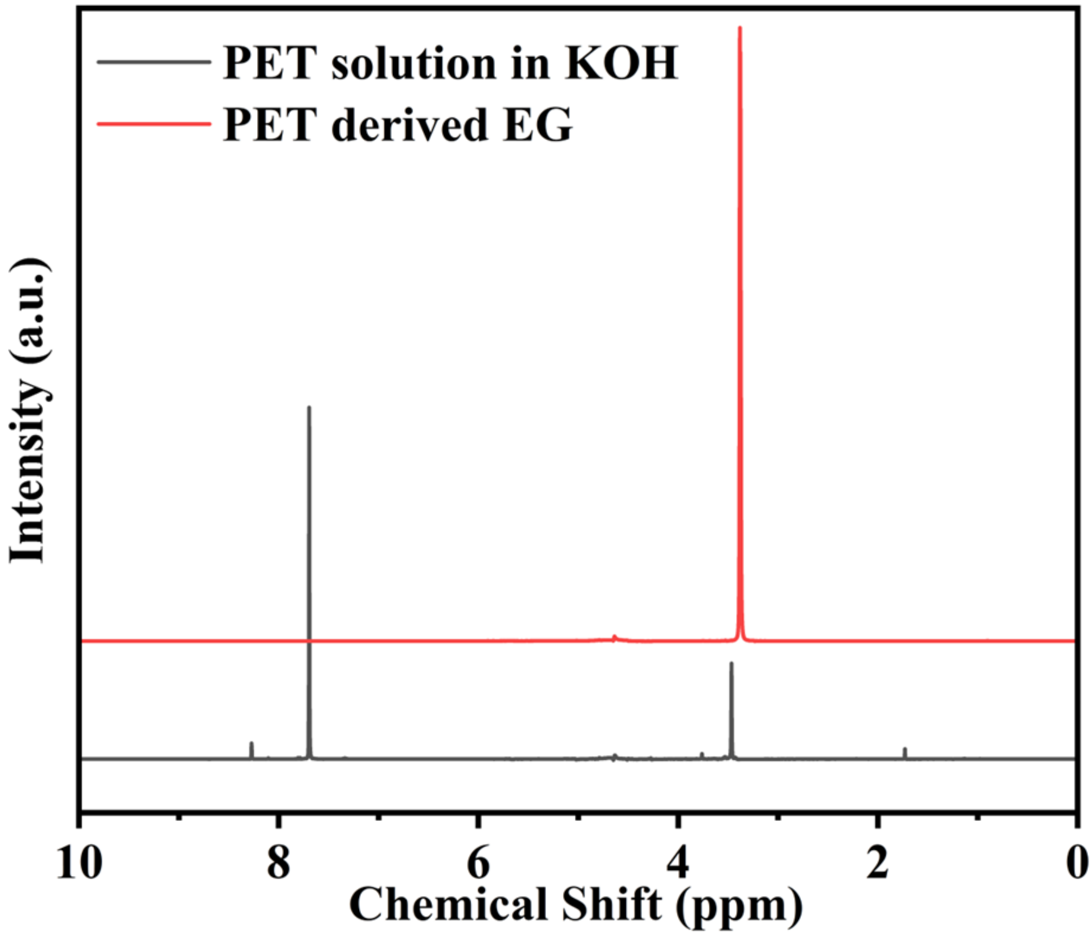

Figure S30. ^{1}H NMR spectrum of a PET solution before (black) and after (red) alkaline hydrolysis in 1 M KOH. The initial PET solution shows characteristic polymer signals, whereas the solution after hydrolysis exhibits peaks at $\delta \approx 3.4$ ppm and other shifts confirming the depolymerization of PET into EG and terephthalic acid as the main products.

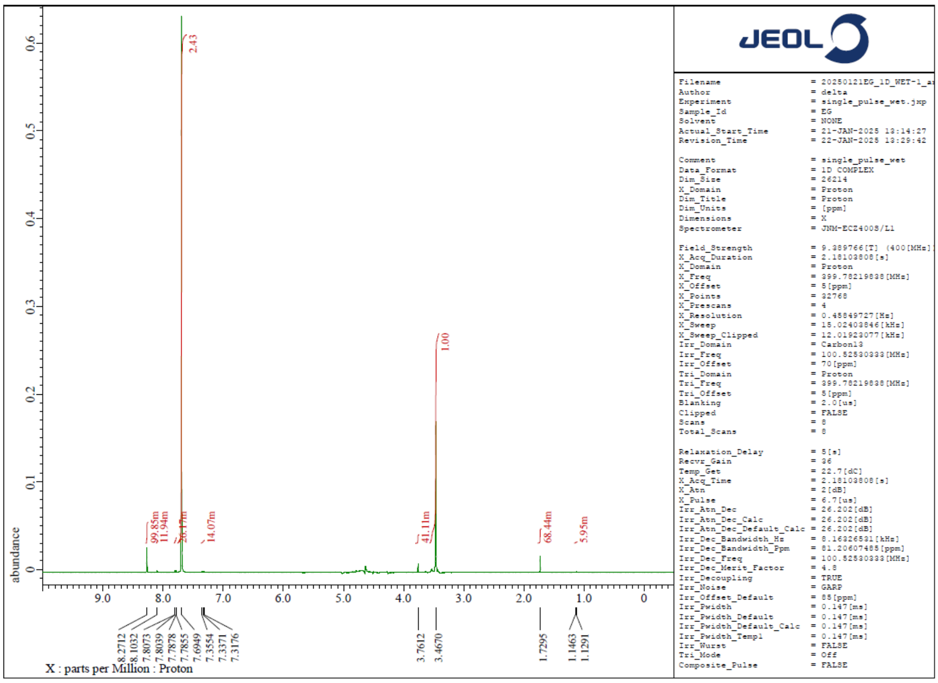

Figure S31. ^{1}H NMR spectrum of the PET solution in 1 M KOH featuring the signals of EG and terephthalic acid as the main products and thus confirming the depolymerization of PET.

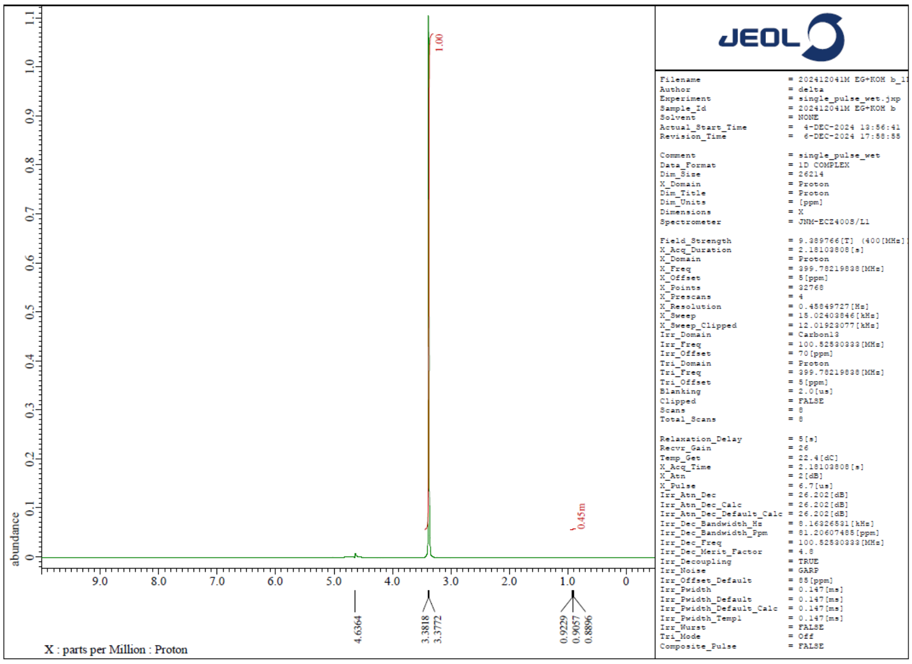

Figure S32. ^{1}H NMR spectrum of the PET solution after alkaline hydrolysis. The characteristic peak at $\delta \approx 3.4$ ppm confirms the formation of EG.

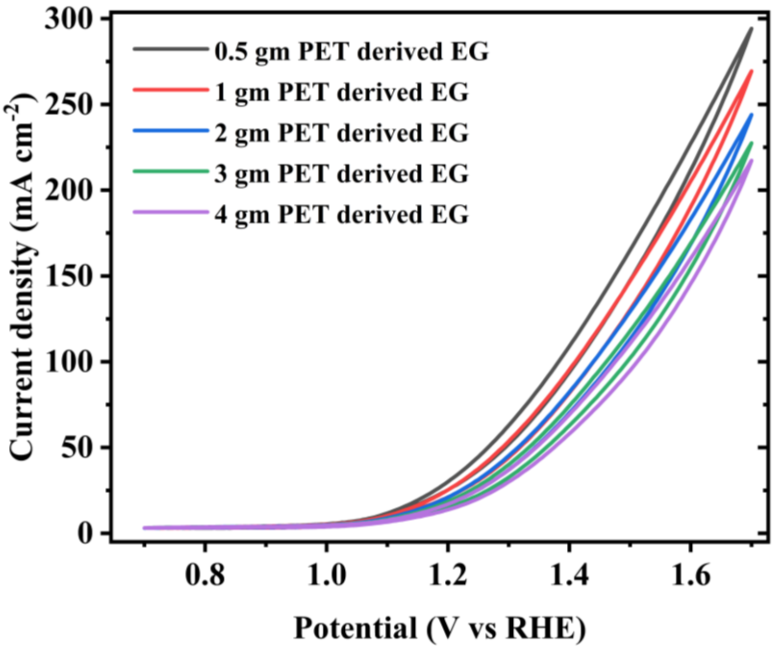

Figure S33. Linear sweep voltammograms of HEOS recorded at varying concentrations of EG in 1 M KOH.

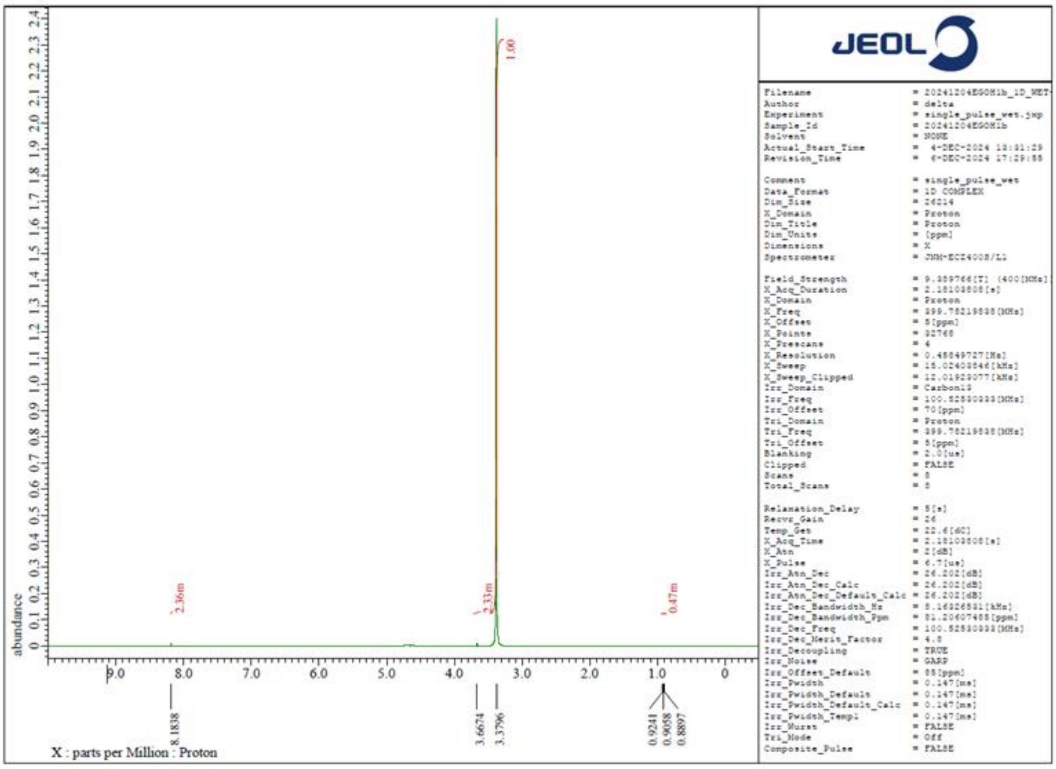

Figure S34. ^{1}H NMR spectrum of the product obtained by the HEOS-catalyzed oxidation of PET-derived EG in 1 M KOH.

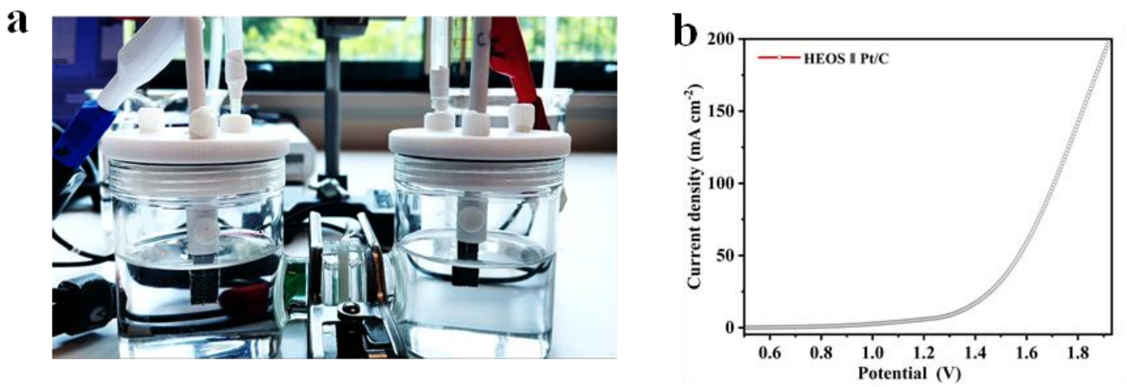

Figure S35. (a) Photograph of the H-type two-electrode setup used for coupled EG oxidation and H_2 evolution, featuring HEOS as the anode material and Pt/C as the cathode material. (b) Polarization curves obtained under various electrolyte conditions, illustrating the electrocatalytic activity and onset potentials for EG oxidation and overall water splitting.

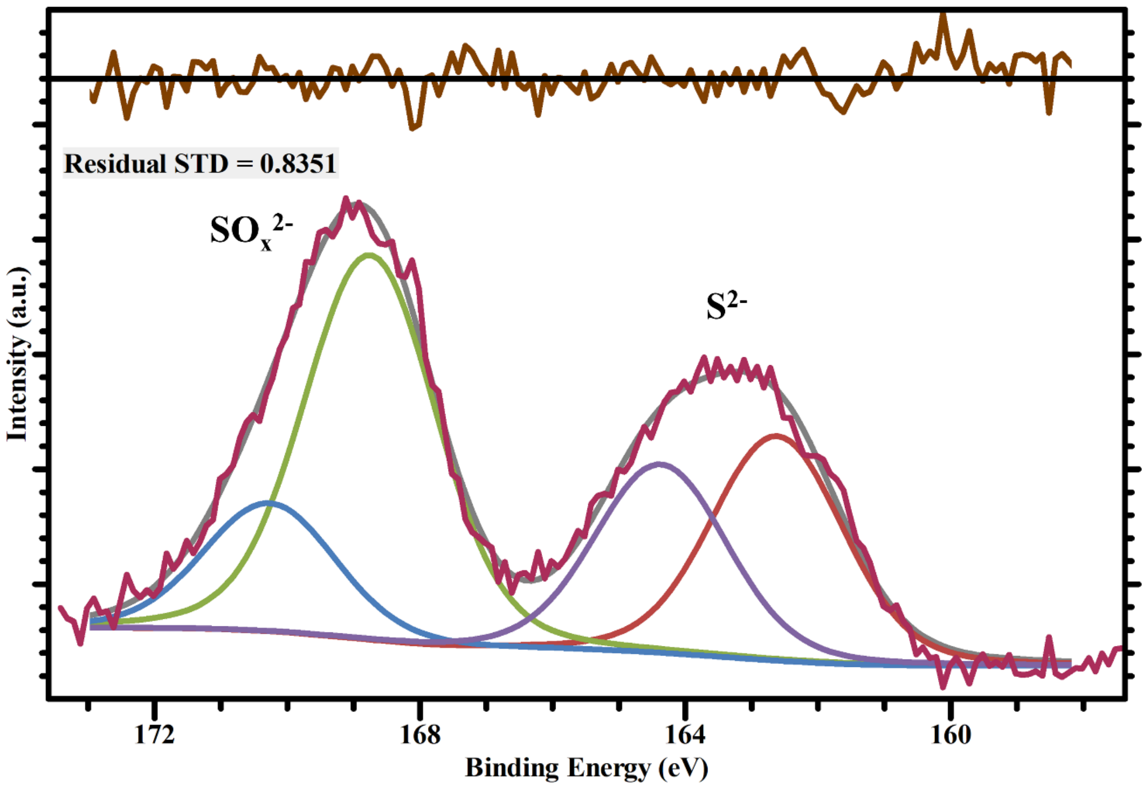

Figure S36. Deconvoluted S 2p X-ray photoelectron spectrum of HEOS after electrochemical testing.

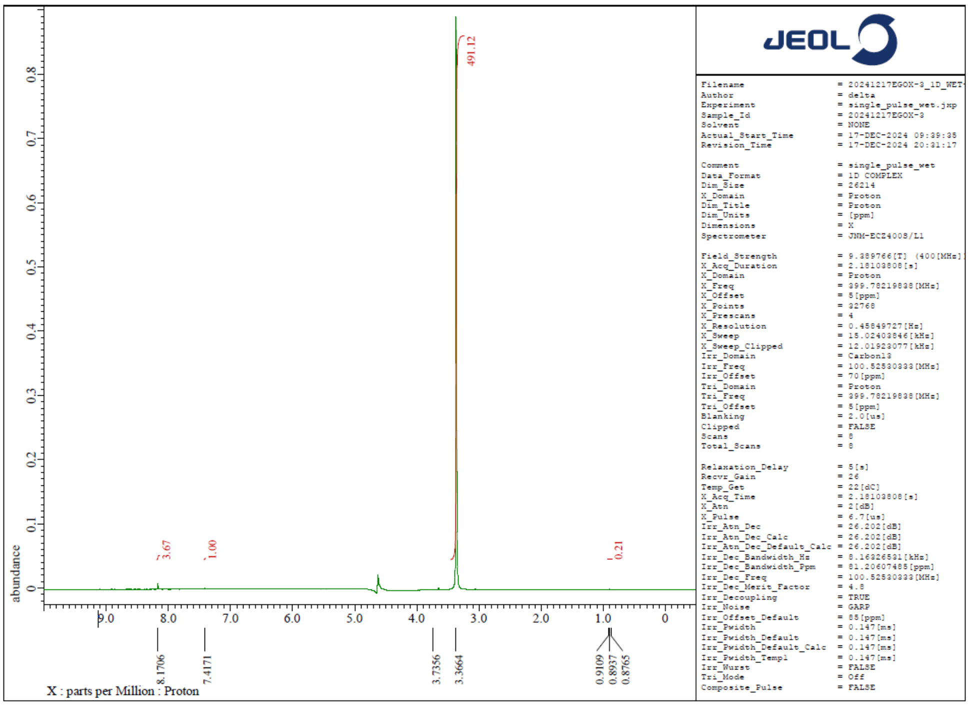

Figure S37. ^{1}H NMR spectrum of the product obtained after 20 h of chronoamperometric testing in a two-electrode setup (electrode surface area = 0.0625 cm^{2}). $CHCl_3$ was used as an internal standard to quantify the product concentration.

References

1. J. Hao, T. Wang, J. Cai et al., “Suppression of structural heterogeneity in high-entropy intermetallics for electrocatalytic upgrading of waste plastics,” Angew. Chem. Int. Ed. 2024, 64, e202419369. https://doi.org/10.1002/anie.202419369
2. Y. Ma, L. Li, J. Tang et al., “Electrochemical PET recycling to formate through ethylene glycol oxidation on Ni–Co–S nanosheet arrays,” J. Mater. Chem. A 2024, 12, 33917–33925. https://doi.org/10.1039/d4ta07156e
3. T. H. Wan, M. Saccoccio, C. Chen, F. Ciucci, “Influence of the discretization methods on the distribution of relaxation times deconvolution: implementing radial basis functions with DRTtools,” Electrochim. Acta 2015, 184, 483–499. https://doi.org/10.1016/j.electacta.2015.09.097

4. A. Sharma, H. Lee, B. Ahn, "Tailoring compressive strength and absorption energy of lightweight multi-phase AlCuSiFeX (X = Cr, Mn, Zn, Sn) high-entropy alloys processed via powder metallurgy," Materials 2021, 14, 4945. https://doi.org/10.3390/ma14174945
5. X. Yang, Y. Zhang, "Prediction of high-entropy stabilized solid-solution in multi-component alloys," Mater. Chem. Phys. 2012, 132, 233–238. https://doi.org/10.1016/j.matchemphys.2011.11.021
6. C. Park, H. Lee, N. Lee, B. Ahn, J. Lee, "Upcycling of abandoned banner via thermocatalytic process over a MnFeCoNiCu high-entropy alloy catalyst," J. Hazard. Mater. 2022, 440, 129825. https://doi.org/10.1016/j.jhazmat.2022.129825
7. A. Sivanantham, H. Lee, S. W. Hwang et al., "Complementary functions of vanadium in boosting electrocatalytic activity of CuCoNiFeMn high-entropy alloy for water splitting," Adv. Funct. Mater. 2023, 33, 2301153. https://doi.org/10.1002/adfm.202301153
8. W. Takahara, "Molecular orbitals of 3d transition metal clusters and their liquid physical metallurgy," Prepr. Natl. Meet. JWS 2013, 93, 164–165. http://ci.nii.ac.jp/naid/10031178316
9. A. Takeuchi, A. Inoue, "Calculations of mixing enthalpy and mismatch entropy for ternary amorphous alloys," Mater. Trans. JIM 2000, 41, 1372–1378. https://doi.org/10.2320/matertrans1989.41.1372
10. T. Matsumoto, M. Sadakiyo, M. L. Ooi et al., "Atomically mixed Fe-group nanoalloys: catalyst design for the selective electrooxidation of ethylene glycol to oxalic acid," Phys. Chem. Chem. Phys. 2015, 17, 11359–11366. https://doi.org/10.1039/c5cp00954e
11. J. Li, L. Li, X. Ma et al., "Selective ethylene glycol oxidation to formate on nickel selenide with simultaneous evolution of hydrogen," Adv. Sci. 2023, 10, 2300841. https://doi.org/10.1002/advs.202300841
12. S. S. Medany, M. A. Hefnawy, "Nickel–cobalt oxides decorated chitosan electrocatalyst for ethylene glycol oxidation," Surf. Interfaces 2023, 40, 103077. https://doi.org/10.1016/j.surfin.2023.103077
13. H. Kang, D. He, X. Yan et al., "Cu promoted the dynamic evolution of Ni-based catalysts for polyethylene terephthalate plastic upcycling," ACS Catal. 2024, 14, 5314–5325. https://doi.org/10.1021/acscatal.3c05509

14. Y. Lin, Y. Chen, H. Ren et al., "Inspiration of bimetallic peroxide for controllable electrooxidizing ethylene glycol through modulating surficial intermediates," Adv. Funct. Mater. 2024, 34, 2404594. https://doi.org/10.1002/adfm.202404594
15. Y. Zhou, Y. Shen, H. Li, "Effects of metallic impurities in alkaline electrolytes on electro-oxidation of water and alcohol molecules," J. Electrochem. Soc. 2021, 168, 124516. https://doi.org/10.1149/1
16. D. Si, B. Xiong, L. Chen, J. Shi, "Highly selective and efficient electrocatalytic synthesis of glycolic acid in coupling with hydrogen evolution," Chem Catal. 2021, 1, 941–955. https://doi.org/10.1016/j.checat.2021.08.001
17. O. O. Fashedemi, H. A. Miller, A. Marchionni, F. Vizza, K. I. Ozoemena, "Electro-oxidation of ethylene glycol and glycerol at palladium-decorated FeCo@Fe core–shell nanocatalysts for alkaline direct alcohol fuel cells: functionalized MWCNT supports and impact on product selectivity," J. Mater. Chem. A 2015, 3, 7145–7156. https://doi.org/10.1039/c5ta00076a
18. H. Wang, Y. Liang, S. Liu et al., "Electron regulation of heterostructured Pt/Rh metallene boosts ethylene glycol electrooxidation and hydrogen evolution," Inorg. Chem. 2023, 62, 14477–14483. https://doi.org/10.1021/acs.inorgchem.3c02487
19. X. Jiang, Z. Dong, Q. Zhang et al., "Decoupled hydrogen evolution from water/seawater splitting by integrating ethylene glycol oxidation on $PtRh_{0.02}$@Rh nanowires with Rh atom modification," J. Mater. Chem. A 2022, 10, 20571–20579. https://doi.org/10.1039/d2ta05469h
20. Z. Niu, S. Fan, X. Li, G. Chen, "Electrocatalytic co-upcycling of nitrite and ethylene glycol over cobalt–copper oxides," Adv. Energy Mater. 2024, 14, 2303515. https://doi.org/10.1002/aenm.202303515
21. I. Bashir, J. D. McGettrick, M. F. Kühnel, B. Sarfraz, S. N. Arshad, A. Rauf, "Sustainable formate synthesis: integrating ethylene glycol oxidation with carbon dioxide electrocatalysis using redox-stabilized earth-abundant electrodes," ACS Sustain. Chem. Eng. 2024, 12, 4795–4802. https://doi.org/10.1021/acssuschemeng.3c08233
22. X. Liu, Z. Fang, D. Xiong et al., "Upcycling PET in parallel with energy-saving H_2 production via bifunctional nickel–cobalt nitride nanosheets," Nano Res. 2022, 16, 4625–4633.

https://doi.org/10.1007/s12274-022-5085-9

23. Z. Xie, K. Wang, Y. Zou, G. Ying, J. Jiang, "Facile surface reconstructions of cobalt–copper phosphide heterostructures enable efficient electrocatalytic glycerol oxidation for energy-saving hydrogen evolution," Energy Adv. 2022, 2, 161–169. https://doi.org/10.1039/d2ya00311b
24. K. Deng, Z. Lian, W. Wang et al., "Lattice strain and charge redistribution of Pt cluster/Ir metallene heterostructure for ethylene glycol to glycolic acid conversion coupled with hydrogen production," Small 2024, 20, 2305000. https://doi.org/10.1002/smll.202305000
